# Characterization of Soaking Process’ Impact in Common Beans Phenolic Composition: Contribute from the Unexplored Portuguese Germplasm

**DOI:** 10.3390/foods8080296

**Published:** 2019-07-28

**Authors:** Elsa Mecha, Susana T. Leitão, Bruna Carbas, Ana T. Serra, Pedro M. Moreira, Maria Manuela Veloso, Ricardo Gomes, Maria E. Figueira, Carla Brites, Maria C. Vaz Patto, Maria R. Bronze

**Affiliations:** 1 NOVA Institute of Chemical and Biological Technology António Xavier, Universidade Nova de Lisboa, Av. da República, EAN, 2780-157 Oeiras, Portugal; 2 INIAV, The National Institute for Agricultural Research and Veterinary, 2784-505 Oeiras, Portugal; 3 Institute for Experimental Biology and Technology, Apartado 12, 2781-901 Oeiras, Portugal; 4 ESAC-IPC, Coimbra College of Agriculture, Polytechnic Institute of Coimbra, 3045-601 Coimbra, Portugal; 5 UniMS-Mass Spectrometry Unit—Institute for Experimental Biology and Technology/ITQB, Av. da República, EAN, 2780-157 Oeiras, Portugal; 6 iMED, Faculty of Pharmacy, Universidade de Lisboa, Av. das Forças Armadas, 1649-019 Lisboa, Portugal

**Keywords:** *Phaseolus vulgaris*, Portuguese varieties, phenolic compounds, soaking, peeling, spectrophotometry, UPLC-TripleTOF-MS

## Abstract

Despite the common beans’ nutritional and phytochemical value, in Portugal its consumption decreased more than 50% in the last decade. The present study aimed to characterize phenolic composition of the Portuguese traditional varieties and corresponding soaked seed fractions (including soaking water). With such purpose, the phenolic composition (total content of soluble phenolics, flavonoids, and proanthocyanidins) and in vitro antioxidant activity were evaluated in the raw whole flour of 31 Portuguese common bean varieties. The phenolic composition of the soaked fractions was respectively compared to the raw flour. Phenolic compounds’ identification and relative quantification were achieved by UPLC-TripleTOF-MS for one representative variety and their fractions. The highest phenolic content was found in colored varieties and the brown market class highlighted as the richest one. The loss of phenolic compounds to the soaking water was highly dependent on variety. The predominant phenolic compounds’ classes were flavan-3-ols (soaking water and coats), flavonols (coats), and phenolic acids (cotyledons). This characterization study showed the diversity on the phenolic composition of Portuguese varieties and the need to adjust the soaking and peeling processes to the variety (considering the possible loss of potential health promoter compounds, e.g., phenolic compounds).

## 1. Introduction

Common bean (*Phaseolus vulgaris* L.) is one of the most widely grown grain legume species (Fabaceae family), cropped across a wide range of different environments from arid climates to humid tropics [1]. Common beans nourish millions of people in developing and developed countries. It fulfills 28% of the carbohydrates, 34% of the dietary fiber and 25% of the protein dietary recommended intake (DRIs) values for an average healthy adult (18–65 years old) [2,3,4]. Common beans are also a rich dietary source of minerals (e.g., magnesium, potassium, zinc, and copper) and vitamins (e.g., vitamins B1, B6, and folate) [4].

Despite the recommendations, nowadays the consumption of common beans, even in the most traditional markets, known by their Mediterranean diet, is decreasing, mainly as a consequence of dietary habit changes [5,6]. In Portugal, regardless of the national rich common bean germplasm [7], data from 2007 to 2017 reported a production decrease of 53%. This decrease is dramatic, considering that common bean represent 75% of the Portuguese grain legumes total consumption and the country is producing less than 10% of its intake, relying heavily on imports [8]. Part of the solution may involve the valorization of traditional varieties in both high and low-income communities.

Grain legume regular consumption has been related to a reduction on the risk of non-communicable diseases (NCD) as cardiovascular diseases, type 2 diabetes *mellitus*, obesity and colon cancer, due to the dietary fiber content and the presence of phenolic compounds [9,10,11] (e.g., phenolic acids and flavonoids as flavanols, flavonols, anthocyanins and isoflavones [12]). 

Several factors, such as genotype, agronomic practices, climatic conditions or harvest conditions (e.g., maturity state), may influence the phenolic composition of common beans [13]. Also, the preparation and cooking processes are critical for the phenolic composition of common beans’ food based products. Domestic preparation may include washing, soaking (previously to thermal processing) and peeling procedures [14]. Soaking is one of the most disseminated procedures at domestic and industrial level, since it softens seeds’ texture, accelerates the cooking process and increases the drained weight [15,16]. Additionally, discarding the soaking water improves seeds’ nutritional quality by removing, at least partially, some anti-nutritional compounds such as the oligossacharides, stachyose and raffinose, (involved in intestinal gas production) [17], phytic acid and tannins (linked to protein and carbohydrates digestibility impairment) [18], saponins (which may cause bloating symptoms and change cholesterol metabolism, by reducing total and LDL cholesterol without changing HDL cholesterol) and trypsin inhibitors (associated to reduced protein digestibility in monogastric animals) [19]. However, processing without discarding the soaking water may preserve these anti-nutritional compounds, which consumption can be advantageous in the context of NCD (e.g., obesity, hypercholesterolemia, cardiovascular diseases) prevention [20].

Despite the highly genetically and morphologically diverse germplasm [7], the phenolic composition of the Portuguese varieties has been unexplored and so far, no comprehensive characterization focused in the different common bean fractions (soaked coats, soaked cotyledons, and soaking water) is available. Although there are several studies regarding the impact of preparation and cooking on the nutritional and protein quality of legumes [21], the effect of soaking process in the phenolic composition is barely known [22] and only a few studies have been dedicated to the distribution of phenolic compounds in the soaked seeds’ fractions (coats and cotyledons) [22].

This study was designed to evaluate the richness of the national common bean genetic resources on phenolic compounds and the effect of the soaking process on the seeds’ phenolic composition. The characterization of these bioactive compounds may provide useful information to breeders, in order to ameliorate the commercially available varieties, as well as to elucidate consumers about the importance of consuming this legume in a daily diet. Moreover, a deeper characterization of the phenolic composition in soaked coats, cotyledons, and corresponding soaking water, may contribute to change some gastronomic practices, such as the bean seed peeling, commonly used in some countries [14], in order to increment the access to bioactive phenolic compounds. To attain these objectives a collection of 31 Portuguese common bean traditional varieties, representing different market classes, was studied. Spectrophotometric assays were conducted to access phenolic compounds’ content and in vitro antioxidant activity on common beans whole flour was also evaluated. Mass spectrometry was performed to identify the main phenolic compounds, in a representative common bean variety, before and after the soaking process.

## 2. Materials and Methods 

### 2.1. Chemicals

Folin-Ciocalteu’s phenol reagent, sodium carbonate (99%), catechin (98%), protocatechuic acid, *p*-hydroxybenzoic acid, gentisic acid, *p*-coumaric acid, sinapic acid, ferulic acid, sodium nitrite (97%), aluminium chloride (99.9%), and vanillin (99%) were purchased from Sigma-Aldrich (St. Louis, MO, USA). Procyanidin B2, rutin, epicatechin, kaempferol, quercetin dehydrate, daidzein and genistein were purchased from Extrasynthese (Genay, France). Sulphuric acid (95–97%) and gallic acid (98%) were purchased from Fluka (Seelze, Germany). Sodium hydroxide (98%) was purchased from Merck (Darmstadt, Germany). Absolute ethanol (99.9%) and methanol (99.9%) were purchased from Carlo Erba Reagents (Rodano, Italy). Milli-Q^®^ water (18.2 MΩ.cm) was obtained in a Millipore–Direct Q3 UV System equipment (Molsheim, France).

### 2.2. Plant Material

31 different traditional common bean varieties were collected from local farmers in the central region of Portugal [23] and kept in cold storage at the Research Unit of Biotechnology and Genetic Resources germplasm bank, INIAV, Oeiras, Portugal (PRT 005). These varieties were multiplied, in 2010, at ESAC (Coimbra, Portugal) under the same edaphoclimatic conditions, using traditional agronomic procedures for common bean production. Information relative to the collection data (geographical location–latitude, longitude, seed color and pattern) is presented in Table 1.

**Figure 1:**
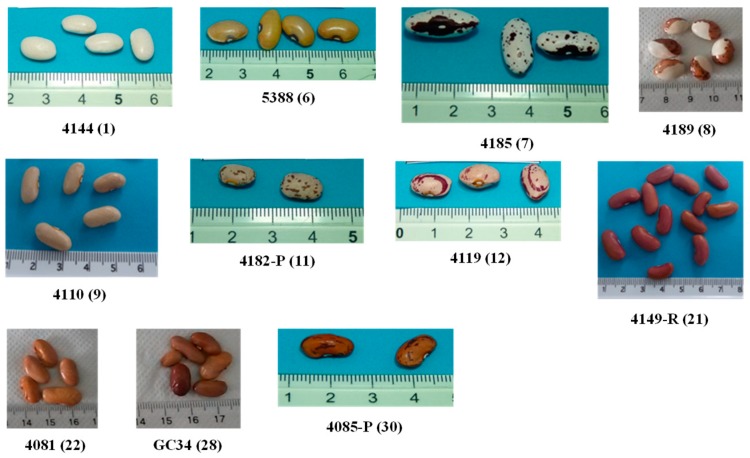
Example of (*Phaseolus vulgaris* L.) common beans varieties morphological aspect (one variety from each color market class).

Seed color and pattern classes (market classes) were defined visually based on *P. vulgaris* plant data descriptors [24]. In Figure 1 one example of each studied common bean market class is shown.

### 2.3. Sample Preparation

#### 2.3.1. Whole Seed Flour Extracts

Dry mature seeds were milled (Falling n° 3100, Perten Instruments, Hägersten, Sweeden) to a particle size of 0.8 mm and stored at −20 °C, until analysis. Extracts were prepared according to Lin et al. (2008) [25] with slight modifications. Briefly, one gram of dry whole seed flour was extracted with 20 mL of methanol: water (60:40, *v*/*v*) solution, followed by sonication, during 60 minutes. The mixture was centrifuged at 420× *g* during 15 min. The volume was adjusted to 20 mL. Final extract was filtered through a 0.45 µm 13 mm PVDF syringe filter (GE Whatman^TM^, Marlborough, MA, USA). Before analysis by UPLC-TripleTOF MS, 5 mL of the extracts were concentrated, until dryness, in a SpeedVac (Labconco, Kansas City, MO, USA) and reconstituted in 1 mL of methanol: water (60:40, *v*/*v*). Extracts were filtered through a 0.20 µm 13 mm CA syringe filter (LLG-Labware, Meckenheim, Germany) and kept at −20 °C, until analysis.

#### 2.3.2. Soaking Water 

Soaking process was performed according to AACC (2007) [26]. Briefly, 100 dry mature seeds were soaked overnight, for 16 h, in distilled water, on the proportion of 1 g per 3 mL of water. Soaking waters were collected, filtered through a 0.45 µm 13 mm PVDF syringe filter (GE Whatman^TM^, Marlborough, MA, USA).

Before analysis by UPLC-TripleTOF MS, 5 mL of the extracts were concentrated, until dryness, in a SpeedVac (Labconco, Kansas City, MO, USA) and reconstituted in 1 mL of Milli-Q^®^ water. Extracts were filtered through a 0.20 µm 13 mm CA syringe filter (LLG-Labware, Meckenheim, Germany) and kept at −20 °C, until analysis.

#### 2.3.3. Coats and Cotyledons Extracts

After soaking, coats were manually separated from cotyledons and both fractions were dried (Memmert^®^ drying oven, Schwabach, Germany) at 30 °C, for an average period of 48 h. Dried coats (0.12 g) were extracted without previous milling, and dried cotyledons were grounded in a mill (Falling n° 3100, Perten Instruments, Hägersten, Sweeden) with mesh 0.8 mm to obtain cotyledons’ flours. The same extraction procedure applied for the common bean whole flour (Section 2.3.1) was applied to cotyledons’ flour (0.88 g). Dried coats were extracted with the methanol: water (60:40, *v*/*v*) solution and the mixture was ground, for 5 min, using a T25 Ultra-turrax equipment (IKA^®^-Werke, Staufen, Germany) followed by sonication during 60 min. All extracts were prepared as triplicates and filtered through a 0.45 µm 13 mm PVDF syringe filter (GE-Whatman^TM^, Marlborough, MA, USA). Before analysis by UPLC-TripleTOF MS, 5 mL of the extracts were concentrated, until dryness, in a SpeedVac (Labconco, Kansas City, MO, USA) and reconstituted in 4 mL of methanol: water (60:40, *v*/*v*). Extracts were filtered through a 0.20 µm 13 mm CA syringe filter (LLG-Labware, Meckenheim, Germany) and kept at −20 °C, until analysis.

### 2.4. Determination of the Phenolic Compounds Content

Total Phenolic Content (TPC), Total Flavonoid Content (TFC) and Total Proanthocyanidin Content (TPAC) were determined as triplicates in the whole flour, as well as in the different fractions (soaking water, whole flour, soaked cotyledons and coats). The content of phenolic compounds from cotyledons and seed coats were expressed per gram of common bean’s dry weight (DW), taking into account the average percent weight of cotyledons (88%) and seed coats (9%) referred to the total seed weight [27]. Seed’s dry weight was determined based on the moisture content (%) assessed by Near Infrared (NIR) analyzer (MPA; Bruker, Billerica, MA, USA), using the flour calibrations for grain legumes provided by Bruker [28].

#### 2.4.1. Total Phenolic Content (TPC)

TPC was determined by the method described by Stamatakis et al. (2009) [29], with modifications. Briefly, Folin-Ciocalteu reagent (0.100 mL) was added to 3.5 mL of extracts previously diluted according to the fraction and variety. After 3 min, 0.400 mL of sodium carbonate solution (35%, *w*/*v*) was added, and after one hour the absorbance was measured against water, in a Spectrophotometer DU-70 (Beckman^®^, Brea, CA, USA), at 725 nm. Gallic acid was used as the external standard in a concentration range of 1 to 6 mg/L of gallic acid. Results were expressed in milligrams of gallic acid equivalents (mg GAE) per g of seed’s dry weight (DW).

#### 2.4.2. Total Flavonoid Content (TFC)

TFC was determined by the method described by Çam & Hışıl (2010) [30]. Briefly, 1 mL of the extract previously diluted or concentrated, depending on the fraction and variety, was added to 4 mL of Milli-Q^®^ water and 0.300 mL of sodium nitrite (5%, *w*/*v*). The mixture was shaken and after 5 min, 0.300 mL of aluminum chloride (10%, *w*/*v*) and 2 mL of 1 M sodium hydroxide solution were added. The final volume (10 mL) was completed with Milli-Q^®^ water. Absorbance was measured against water, in a Spectrophotometer DU-70 (Beckman^®^, Brea, CA, USA), at 510 nm. (+)-Catechin was used as the external standard in a concentration range of 20 to 100 mg/L. Results were expressed in mg of (+)-catechin equivalents (mg CE) per g of seed’s dry weight (DW).

#### 2.4.3. Total Proanthocyanidin Content (TPAC)

TPAC was determined by the vanillin assay following Çam & Hışıl (2010) [30] with modifications. Briefly, extracts and soaking water were concentrated, until dryness, in a SpeedVac concentrator (Labconco, Kansas City, MO, USA). Methanol (5 mL) was added to dried samples, and after shaking in vortex, 1 mL of the supernatant was mixed with 2.5 mL of vanillin 1% in methanol and 2.5 mL of H_2_SO_4_ 25% in methanol. The mixture rested during 15 min at 30 °C and the absorbance measured against methanol in a Genesys 10UV Spectrophotometer (Thermo Spectronic^TM^, ThermoFisher Scientific^®^, Waltham, MA, USA), at 500 nm. (+)-Catechin was used as the external standard in a calibration range of 2.5 to 100 mg/L. Results were expressed in mg of (+)-catechin equivalents (mg CE) per g of seed’s dry weight (DW).

### 2.5. In Vitro Antioxidant Activity

The Oxygen Radical Absorbance Capacity (ORAC) assay was applied to evaluate antioxidant capacity of common bean whole flour towards peroxyl radicals. The assay was carried out following a modified method described by Ou et al. (2001) [31], in order to measure the ability of antioxidant species, present in the sample, to inhibit Fluorescein (FL) oxidation catalyzed by 2,2’-Azobis(2-amidinopropane) dihydrochloride (AAPH)—generated peroxyl radicals (ROO^.^). The reaction mixture included 6.3 × 10^−8^ M FL, 1.28 × 10^−2^ M AAPH (prepared in 75 mM PBS, pH 7.4) and the diluted sample, in a total volume of 1.8 mL. The reaction started by addition of AAPH to the mixture, placed in a 10 mm wide fluorescence cuvette at 37 °C. Fluorescence emitted by the reduced form of FL was measured and recorded every 1 min at the emission wavelength of 515 nm and excitation wavelength of 493 nm (fluorescence spectrophotometer with thermostatic bath, model Cary Eclipse, Varian Ltd., Surrey, UK) for a period of 30 min. PBS was used as blank and 1, 5, 12.5, 25 and 50 M Trolox solutions as control standards. For ORAC analysis only the whole flour extracts were analyzed. All samples, including blank and controls, were analyzed in triplicate. Final ORAC values were calculated using a regression equation established between Trolox concentration and the net area under FL decay curve. Data were expressed in micromoles of Trolox equivalents antioxidant capacity (TEAC) per g of seed’s dry weight (DW).

### 2.6. Phenolic Compounds Identification and Relative Quantification

One representative variety with the highest qualitative diversity of phenolic compounds was selected and characterized using a UPLC-TripleTOF 6600 mass spectrometer. Additionally, relative quantification of the identified compounds was also performed.

#### Analysis by UPLC-TripleTOF 6600 Mass Spectrometer

The UPLC analysis was carried out on UPLC Acquity (Waters, Milford, MA, USA). The chromatographic separation was performed on a LiChrospher^®^ 100 RP18 (250 × 4 mm, particle size 5 µm, Merck, Darmstadt, Germany) thermostated at 35 °C. The injection volume was 20 µL. The flow rate was set at 300 µL/min. The mobile phase was composed by eluent A (0.5% formic acid + 95.5% MQ^®^ Water) and eluent B (90% acetonitrile + 9.5% MQ^®^ water + 0.5% formic acid). Initially, an isocratic condition, corresponding to 5.6% of eluent B was used for 10 min, followed by a gradient elution, 16.7% of eluent B for 30 min and 22.2% of eluent B at 45 min. The percentage of eluent B remained at 22.2% for 20 min followed by an increase to 60.0% at 95 min and to 70.0% at 110 min. The initial elution conditions were established (5.6% of eluent B) during 20 min. The UPLC system was coupled to a TripleTOF 6600 mass spectrometer (SCIEX, Framingham, MA, USA). The system was tuned using the taurocholic acid solution 2 ng/µL (Ref: 44052241) and calibrated with the ESI negative calibration solution (Ref: 4463277), both solutions from SCIEX.

The detection was performed in a mass range of *m*/*z* 50.0–1000.0. Samples were analyzed in the negative mode with a capillary voltage of +4500 V, using Curtain GasTM at 30 psi, Gas1 (nebulizer gas) at 60 psi and Gas2 (heater gas) at 50 psi. Samples were vaporized at 500 °C. Information Dependent Acquisition mode (IDA) was used to select the 20 most intense ions, with intensity greater than 100 cps. For MS^2^ experiments it was applied collision energy of −25 V, with a collision energy spread of 15 V. The dynamic background subtraction was chosen. The MS and MS^2^ data were processed in PeakView 2.1 Software (SCIEX, Framingham, MA, USA). When possible, the identification of phenolic compounds was performed based on the comparison of the retention time, fragmentation pattern and mass accuracy of available commercial standards. Since for the majority of compounds there are no commercial standards, the tentative identification was based on the fragmentation pattern, accurate mass measurements (mass error ≤ 5 ppm) and comparison with available literature on phenolic compounds.

The relative quantification was expressed per each fraction analyzed as Compounds’ class area = Compounds’ class area/Total quantified area and % Area (each compound in the class) = Compounds’ area/Total quantified compounds’ class area, which allowed comparison of the different common bean’s fractions, before and after the soaking process, considering the relative abundance of each compounds’ class, as well as the abundance of each identified compound in the corresponding phenolic compounds’ class.

### 2.7. Data Analysis

All data statistical analyses were conducted in IBM^®^ SPSS^®^ Statistics, version 22, Armonk, NY, USA. ANOVA test was performed for each parameter analyzed in the extracts, after testing each parameter for the normality (Shapiro-Wilk test). Variables transformation by logarithmic or two-step transformations [32] were performed when the variables were not normally distributed. Multivariate analysis was performed by Principal Component Analysis (PCA) followed by k-means cluster analysis to classify common bean traditional varieties (*n* = 31) based on the PCA solution. The color and pattern of common bean varieties were superimposed to the distribution of the samples on the bi-dimensional space defined by the two first principal components. The number of clusters was established by comparison of the determination coefficient (R^2^) obtained for different clusters’ solutions (K = 2 to K = 6) and the clustering solution confirmed by discriminant analysis. The groups defined by cluster analysis were compared by ANOVA test and significant differences between groups determined by the post hoc Scheffé’s test or by the Games–Howell test (depending, respectively, on the acceptance or violation of the homoscedasticity criterion) at a significance level of 5%. Although the multivariate analysis had been developed with some transformed variables, the clusters’ characterization was reported taking in account the corresponding results on the non-transformed (original) variables. Correlations between quantitative variables were defined by Pearson’s R coefficient [33].

## 3. Results and Discussion

### 3.1. Phenolic Content in Common Beans’ Whole Flour, Before Soaking

Phenolic compounds are bioactive molecules that are found in common beans samples. Table 2 presents a summary of the results obtained for the whole flour before the soaking process, for the different fractions (coats and cotyledons), after the soaking, as well as for the soaking water. Samples were organized into different color classes, according to seeds’ color similarity. As only one yellow variety was studied, the results from this sample were excluded from Table 2.

As expected, since some of these compounds are known for their contribution to seed color [39], the colored market classes were richer in phenolic compounds (including flavonoids and proanthocyanidins). A strong positive correlation was found between TPC in whole flour extracts and TFC (Pearson’s R of 0.890, *p* < 0.05), as well as between TPC and TPAC (Pearson’s R of 0.746, *p* < 0.05), as already described by Nyau et al. (2016) [35]. For the white (*n* = 5) and brown varieties (*n* = 10) studied, Table 2, the average TPC values were higher than the ones previously described for the brown colored Zambian varieties [35].

The same was verified for the red varieties (*n* = 5) with TPC, TFC and TPAC average values higher than the ones, previously, reported for a red kidney market class [37]. The differences observed between the measured phenolic content and the values described in the literature [35,37] can be attributed to different causes, such as common beans’ genotypes and geographical origins but, also, to differences in the extraction protocol. Although the extracting solvent used herein and in literature [35,37] was methanol, there were differences in the proportion of solvent per mass of common beans’ flour [35,37], as well as in the extraction technique applied, herein with ultra-sonication and in Xu et al. (2007) [37] with orbital shaker.

In the present study, and among the brown varieties, the ones identified as 27, 30 and 31 showed the highest TPC values, contributing for the high variability of such market class (coefficient of variation, 24%). In what concerns the TFC and TPAC values, this market class also showed high variability in TFC and TPAC values (coefficient of variation, 35%, and 60%, respectively). From the results obtained, the brown market class was considered a highly valuable class for future breeding programs focused on increasing phenolic compounds content. The role of phenolic compounds in the prevention of chronic diseases has been attributed to their free radicals’ scavenging ability [40] responsible by their antioxidant properties. Several different chemical assays such as ORAC (oxygen radical absorbance capacity), HORAC (hydroxyl radical antioxidant capacity), DPPH (2,2-diphenyl-1-picrylhydrazyl) and FRAP (ferric reducing antioxidant power assay) have been used to evaluate compounds’ antioxidant activity. The major difference between these assays is the nature of the molecules reduced by the antioxidant compounds. While ORAC measures the ability of antioxidant compounds to reduce the peroxyl radicals, in HORAC the hydroxyl radicals are the reduced compounds. In DPPH the reduced compound is 2,2-diphenyl-1-picrylhydrazyl and in FRAP the complex Fe^3+^ tripyridyltriazine is the reduced one [41].

In the present study, the ORAC method was the selected one, since previous works showed higher correlation with TPC of legumes’ extracts [34]. Since the variation between the TPC triplicate values, determined in the whole flour extracts, ranged between 0.7 and 6.6% for ORAC determination, only one extract per variety was randomly analyzed. As shown in Table 2, the pink, red and brown varieties revealed the highest ORAC values, 143.22 ± 35.39, 125.46 ± 31.50 and 154.83 ± 40.41 μmol TEAC/g DW, respectively, and the white varieties the lowest, 37.35 ± 4.77 μmol TEAC/g DW, following the same trend of the phenolic content. These values were higher than the ones obtained for pink and red varieties described by Xu et al. (2007b) [42], Table 2. ORAC values also presented a strong correlation with TPC and TFC values, Pearson’s R of 0.909 and 0.918, respectively.

Considering that all the common bean varieties were cultivated under the same edaphoclimatic conditions, the variability found in the phenolic compounds must be strongly dependent on the common bean variety.

### 3.2. Phenolic Content in Soaking Water, Soaked Coats and Soaked Cotyledons

In the beans’ processing industry, soaking water has been traditionally discarded, remaining one of the most underexplored byproducts. The results obtained in this work showed that the percentage of TPC in the soaking water can vary a lot with the common bean variety, regardless of the phenolic content in the raw whole beans. While in a white variety soaking water’s TPC represented 5–14% of the TPC in the whole flour, for brown varieties the values ranged from 28 to 55% and the highest value, 66%, was determined in the light brown variety (sample 22), Table S1. The results obtained, herein, contradict previous results reported for the soaking water of black beans and cream background beans with pink and red stripes [43], described with minor phenolic compound losses <2% [13] or with no quantifiable phenolics in soaking water [43].

The strong positive correlation between TPC and TFC (Pearson’s R: 0.965, *p* < 0.05), in the soaking water is due to the leaching process of highly soluble flavonoids from coats into water. The variability found in the phenolic compounds’ loss (including flavonoids and proanthocyanidins) into the soaking water is strongly dependent on the common bean variety and particularly on seeds’ permeability to water [44] and seeds’ dormancy-breaking regimes [45,46]. Some varieties had TPAC losses into the soaking water lower than 25% (e.g., sample 28) and for other varieties (e.g., samples 1 to 5, 16, 18, 19 and 24) the phenolics determined in the raw seeds were completely lost into the soaking water, which might be related with a high rate of water uptake, typical of varieties with “soft” coats [44]. Despite the expected high loss of phenolic compounds, these “soft” coat” varieties are valuable for food industry and consumers focused on reducing the cooking time duration required to process the beans.

After the soaking process, on average, soaked beans showed higher phenolic content (including flavonoids and proanthocyanidins) than the non-soaked whole flour. These results may be attributed to the extraction of soaked coats and soaked cotyledons, performed separately after the soaking process. Such procedure allowed a higher extraction rate of the free phenolic compounds present in coats fraction, since cotyledons removal could eliminate some phenolic-protein interactions [47]. For this reason, despite the phenolic compounds’ loss into the soaking water, an overall comparison of common beans TPC in whole flour and in soaked beans (calculated as the sum of the TPC determined, separately, in both soaked fractions, coats and cotyledons) showed that at least 51% of the TPC determined in non-soaked seeds was preserved after soaking, which contradicts the high decrease, −73%, in the TPC of soaked beans reported by Faller & Fialho (2012) [48].

The comparison study performed between the phenolic content determined in cotyledons and coats, Table S2, revealed that for soaked white beans, cotyledons fraction had higher contribution to TPC (95–96%) and TFC (94–96%) than coats, which is in accordance to Sutivisedsak et al. (2010) [38]. By opposition, in colored varieties, the coats revealed higher phenolic and flavonoid contents than the cotyledons, which support the consumption of the colored common bean seeds without peeling as a strategy to preserve common beans’ phenolic compounds. The results suggested that the peeling process, traditionally performed in some African countries to prepare porridge and recipes like Akara, Moin Moin and Gbegiri soup, might impair the phenolic content of the final common bean food-based products. Therefore, and bearing in mind the impact of the peeling process on the nutritional food quality (by enhancing protein and carbohydrate digestibility) but also on the elimination of compounds with potential health effect [14], the traditional recipes could be adjusted and reinvented to include or not the peeling process, depending on the populations’ nutritional status.

### 3.3. Phenolic Compounds’ Characterization

Although the information regarding the total phenolic content has been fundamental to characterize beans’ samples, analysis of individual compounds has become mandatory and for that separation methodologies must be used. The individual study of phenolic compounds is challenging as the samples are complex (with a large number of compounds at different concentration ranges) and often there are no commercially available standards. Presently the identification of individual compounds is achieved mostly by mass spectrometry associated to chromatographic techniques.

**Figure 2:**
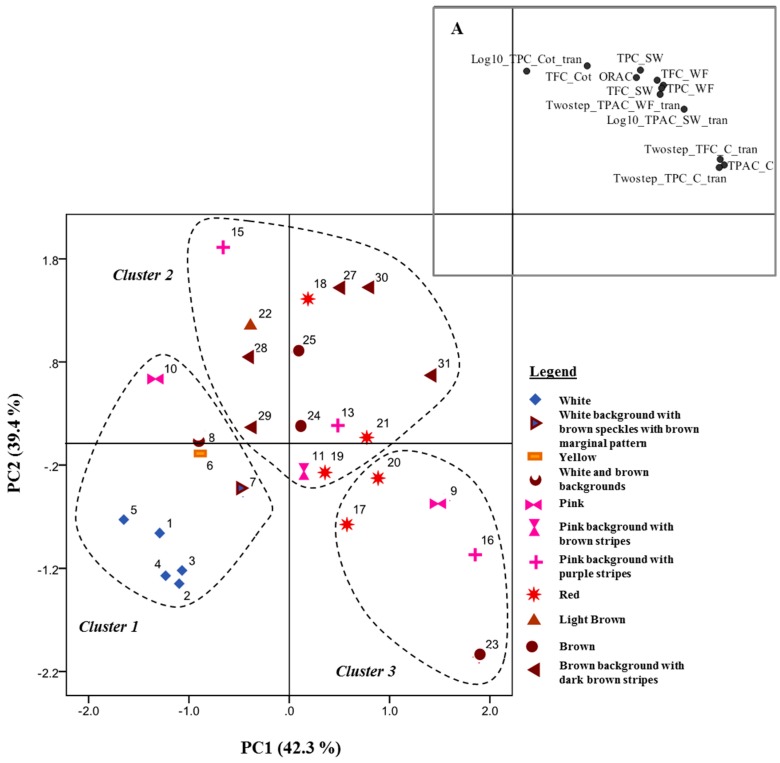
Projection of the Portuguese common bean varieties (n = 28) in a bi-dimensional space (Principal Component 1—PC1 and Principal Component 2—PC2). Color of samples was depicted and clusters highlighted. Explanation for subfigure **A**—Distribution of the parameters analyzed in the bidimensional space (TPCTotal Phenolic Content; TFC—Total Flavonoid Content; TPAC—Total Proanthocyanidin Content; ORAC—Oxygen Radical Absorbance Capacity; C—Coats; Cot—Cotyledons; SW—Soaking Water; WF—Whole Flour). To achieve normal distribution, the parameters TPC in Cot and TPAC in SW were submitted to the logarithmic (Log_10_) transformation and the parameters TPC in C, TFC in C and TPAC in WF to the two-step transformation, before multivariate analysis. The two-step transformation excluded from analysis samples 12, 14 and 26, since TPC_C, TFC_C and TPAC_WF were out of ranking.

In this work, one representative variety, with high qualitative diversity of phenolic compounds, was selected to identify the individual phenolic compounds present in common bean. The selection was done after the PCA analysis of the measured global parameters, followed by cluster analysis, Figure 2 and Table S3. This procedure allowed classifying common bean samples into three different clusters, Figure 2 and Table S4, which explained 65.9% of the total variance.

The discriminant analysis confirmed the clustering solution, Table S5. All the clusters revealed high variability in the phenolic content, Table S4. Sample 22, a light brown variety, chosen as a representative variety, was located in cluster 2, in an intermediate position between cluster 1 (characterized by the samples with the lowest phenolic content) and cluster 3 (characterized by the samples with the highest phenolic content in coats’ fraction).

The different fractions of the representative variety were analyzed by UPLC-Triple-TOF-MS, in negative mode, for compounds’ identification and relative quantification, as presented in Table 3 and Table 4. Identification was based on the retention time, mass accuracy, fragmentation pattern and previous description in literature.

**Figure 3:**
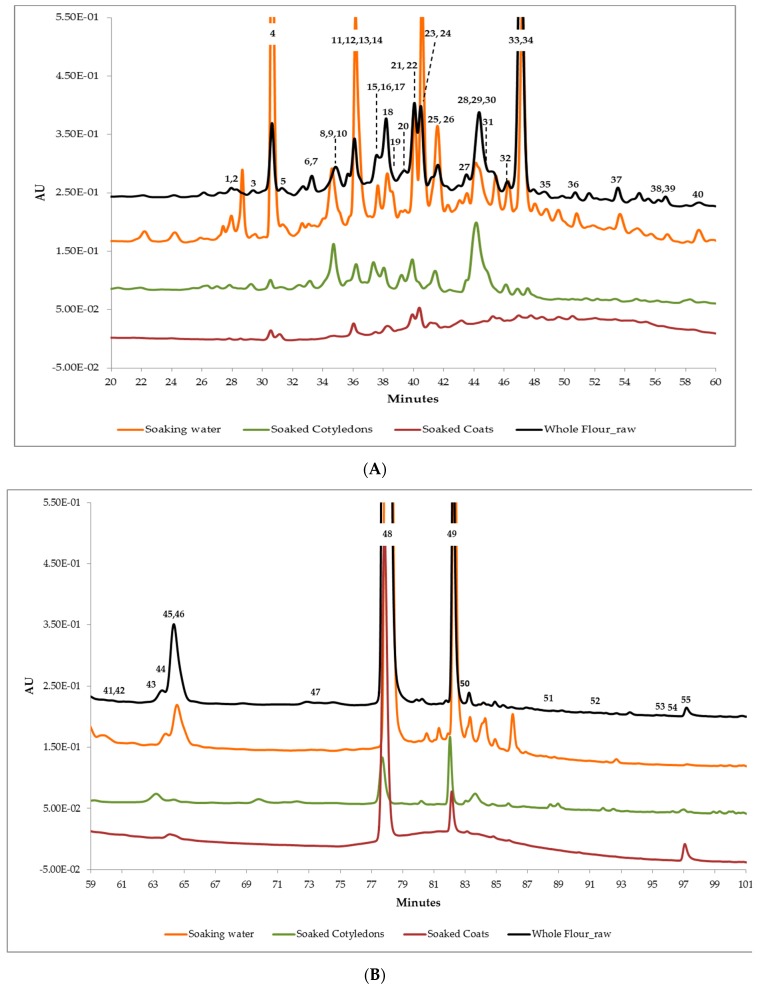
(**A**) Zoom of the chromatographic profiles of sample’s 22 fractions (20–60 min) at 280 nm. The numbers highlight the identified compounds in Table 4. (**B**) Zoom of the chromatographic profiles of sample’s 22 fractions (59–101 min) at 280 nm. The numbers highlight the identified compounds in Table 4.

The chromatographic profiles of the different fractions, at 280 nm, are compared in Figure 3A,B.

**Figure 4:**
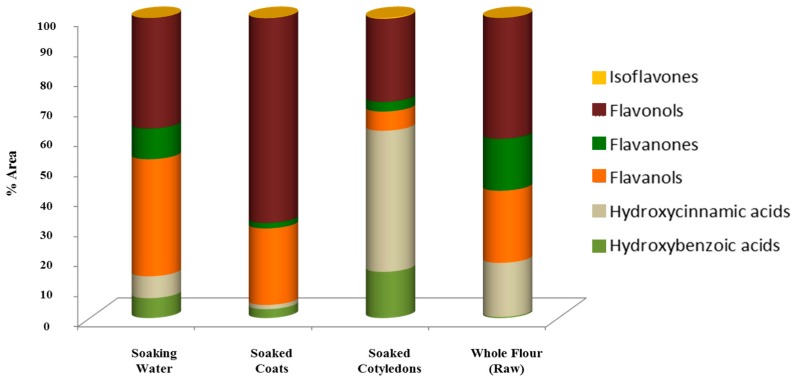
Percentage, %, area of compounds’ classes in different sample’s 22 fractions.

Compounds were classified in six different phenolic compounds’ classes: hydroxybenzoic acids, hydroxycinnamic acids, flavan-3-ols, flavanones, flavonols, isoflavones, and their structures are shown in supplementary material, Table S6. The relative quantification was expressed as the % area of the compounds’ class area considering the total area, Figure 4, and the % area of the identified compound considering the total compounds’ class area, Table 4.

#### 3.3.1. Hydroxybenzoic Acids

Hydroxybenzoic acids’ biological activity on the endothelial dysfunction, blood lipid profile and inflammation indicates their important role in cardiovascular system, as reviewed by Juurlink et al. (2014) [63]. These compounds were identified in all common bean fractions analyzed, representing from 0.4% of the total identified compounds, in raw whole flour, to 14.7% of the total identified compounds, in soaked cotyledons.

Among hydroxybenzoic acids, identified in the present study, vanillic acid (2) was mostly abundant in the raw flour and its presence was previously described in dark beans [50]. The soaked coats represented the richest fraction in protocatechuic acid, which was already identified by Xu & Chang (2009) [52] and López et al. (2013) [53] in dark, pinto and black beans. Protocatechuic acid-4-*O*-glucoside (1) and *p*-hydroxybenzoic acid-4-*O*-glucoside (12) were also identified based on the fragmentation experiments, which showed the product ions *m*/*z* 153.0229 and *m*/*z* 137.0304, corresponding to protocatechuic acid and *p*-hydroxybenzoic acid, respectively. The presence of the glycosidic forms of protocatechuic and *p*-hydroxybenzoic acids in legumes was described, for the first time, by Moran et al. (1997) [49] in the soybean root nodules. Gentisic acid (27) was identified using an external standard, and is known to be widely distributed in legume species [13,54]. Because of its water solubility, the glycosidic forms of protocatechuic and *p*-hydroxybenzoic acid, as well as gentisic acid were dominant in soaking water.

#### 3.3.2. Hydroxycinnamic Acids

Hydroxycinnamic acids were mostly present in soaked cotyledons (49.3% of the total identified compounds), as shown in Figure 4, and included the *p*-coumaric (41), sinapic (44) and ferulic acid (45), as well as their aldaric derivatives.

As described by Aguilera et al. (2011) [51] and Dueñas et al. (2015) [55], the aldaric derivatives of hydroxycinnamic acids represent the most typical hydroxycinnamic acids in common beans. In the selected light brown variety it was possible to identify, mostly in soaked coats, six isomers of *p*-coumaroyl aldaric acid, seven isomers of feruloyl aldaric acid and five isomers of sinapoyl aldaric acid. The *p*-coumaric acid derivative, *p*-coumaroyl aldaric acid 6, was not found in soaking water. The free hydroxycinnamic acids, *p*-coumaric and ferulic acids, were mainly detected in the soaking water (3.1% of the total hydroxycinnamic acids), possibly due to the enzymatic disruption of some glycosidic bonds attached to the phenolic acids in the seeds’ cell walls.

#### 3.3.3. Flavan-3-ols

Flavan-3-ols are known as important phenolic compounds in common beans [51], and represented 39.0% and 25.5% of the total compounds identified in soaking water and soaked coats, respectively, Figure 4. As shown in Table 4, the oligomeric forms of flavanols were mostly dimeric and trimeric procyanidins. Although this class of compounds has been assumed as anti-nutritional, nowadays their value has been recognized, not only in the feeding industry, to improve animals’ growth and gut health, acting as a promising alternative to antibiotics in poultry [64], but also to human health, as prebiotic compounds in colon and as protective factors against cariogenic bacteria in the mouth (*Streptococcus mutans*) and ulcerogenic *Helicobacter pylori* in the stomach [65].

In the whole flour of the light brown variety analyzed it was possible to identify 5 dimeric procyanidins, at different retention times. Procyanidins dimmers B2 and B3 were described previously in pinto beans [51] and the dimmer B4 [53] in dark beans. Identification of procyanidin B5 was performed in peanut peel [59] but, as far as we know, this is the first paper that describes the presence of the dimmer B5 in common bean. (+)-Catechin-3′-*O*-glucoside (4) was identified and previously described in pinto beans [51]. On the other hand, (+)-catechin-7-*O*-glucoside (17) was tentatively identified herein, for the first time in common beans, and was previously described in cowpea [56].

Mostly present in seeds’ coat and soaking water, as previously stated by Aguilera et al. (2011) [51], in this compounds’ class, the major compounds identified included (+)-catechin-3′-*O*-glucoside, procyanidin B1, and (+)-catechin. The presence of such compounds in seeds’ coats has been associated to embryos’ protection against pathogens, ensuring seeds’ germination [66].

In the cotyledons’ fraction the most abundant flavan-3-ol was the (+)-catechin-3′-*O*-glucoside. Proanthocyanidins, like procyanidin B1, B3 and B4, were below the detection limit of the method, possibly as a consequence of the crosslinking reactions with proteins, highly accumulated in cotyledons fraction [67] making these compounds not accessible for extraction.

#### 3.3.4. Flavanones

The aglycone forms of naringenin (54) and eriodictyol (51) and their glycosidic forms were accurately identified in all the studied fractions. The aglycones were previously described in whole flour of white beans [51]. Compounds 29, 34, 37 and 38 were identified as eriodictyol-hexoside isomers, since they showed a product ion at *m*/*z* 287.06, which corresponds to the loss of an eriodictyol unit. Aguilera et al. (2011) [51] also described in white beans a deprotonated molecule with *m*/*z* 449 as an eriodictyol derivative. Naringenin-7-glucoside was also previously described [53] in dark beans. In the light brown variety analyzed, this phenolic compounds’ class represented 17.3% and 10.2% of the total phenolic compounds, in whole flour and soaking water, respectively. Eriodictyol-hexoside 2 was the most predominant flavanone in all the fractions (more than 70% of the identified flavanones). The aglycones, eriodictyol and naringenin contributed to 15.1% of the flavanones total area in soaked coats fraction, suggesting that these compounds are mostly located in seeds’ coat rather than in cotyledons.

Recent studies on eriodictyol’s cell activity suggest the potential of such compound in immunomodulation, anti-inflammation and anti-oxidant activity [68], which supports the importance of preserving, on food based-products, common beans coats naturally rich in these flavanones.

#### 3.3.5. Flavonols

Flavonols represent one of the most abundant phenolic compounds’ classes in common beans [25], especially in soaked coats.

In the present study, they represented 68.2%, 40.3%, and 36.9% of the total compounds identified in soaked coats, whole flour before soaking and soaking water, respectively, Figure 4.

Quercetin (52) and kaempferol (55) were identified, as well as their derivatives. The identification of the product ion at *m*/*z* 301.04 in the fragmentation of compound (41) was a clear indication for rutin (quercetin-3-*O*-rutinoside) identification, as described by Lin et al. (2008) [25]. Quercetin glycosides (46 and 47) were also identified in the present study based on the characteristic quercetin product ion, *m*/*z* 301.04, as previously described by Pitura (2011) [62], in colored common bean varieties.

Compound 39 was tentatively identified as luteolin-3′,7-di-*O*-glucoside or as kaempferol-3′,7-diglucoside, considering the characteristic fragment ion at *m*/*z* 285.04. As far as we know, these compounds have not been previously described in common beans, but luteolin-3′,7-di-*O*-glucoside was identified in chickpea [60] and pea [60], and the kaempferol-3′,7-diglucoside in broad beans [69].

Recent research highlighted the role of kaempferol as an antioxidant and immune-modulator agent with potential anti-carcinogenic effect in 5-fluorouracil resistant LS174 colon cancer cells [70]. Such beneficial health impact supports the regular consumption of common bean, including the coats’ fraction, as a natural rich source of kaempferol aglycone (55) (20.0% of the total flavonols’ area). Besides the aglycone form, kaempferol derivatives (46, 49 and 50) were also identified and previously reported in common beans [61]. Kaempferol-3-*O*-glucoside was the most abundant flavonol in soaked coats, soaking water and whole flour, representing, respectively, 66.5%, 53.2% and 44.3% of the total flavonols’ area. In soaked cotyledons the predominant kaempferol derivative was the kaempferol-3-*O*-acetyl-glucoside. Similarly to kaempferol, kaempferol-3-*O*-glucoside has, also, been described as a biological active agent in inflammatory pathological conditions and as a scavenger of free radical compounds, showing anti-cancer activity in several cancer cell lines [71].

#### 3.3.6. Isoflavones

Isoflavones was the less representative class of phenolic compounds found before and after the soaking process. It represented only 0.3% and 0.1% of the total quantified area, in soaked cotyledons and soaked coats, respectively. Daidzein and genistein were the isoflavones identified after the soaking process, in soaking water, soaked coats and soaked cotyledons and have been described previously in yellow and black common bean varieties [61]. Before the soaking process, only daidzein was identified. As water-soluble compounds, isoflavones are present in the soaking water [72]. Nevertheless, this phenolic compounds’ class only represented 0.02% of the total compounds diffused into water, indicating its low contribution to the soaking water’s phenolic content.

## 4. Conclusions

The results obtained in this study showed that the phenolic compounds’ content in the highly genetically diverse Portuguese common bean germplasm was quite variable. Such variability allowed for study of the impact of the traditional soaking process only with water, overnight, in different common bean varieties. During the soaking period, the percentage of phenolic compounds lost into the water was dependent on common bean’s variety. Therefore, the soaking process should be adapted to each variety in accordance to the populations’ nutritional needs. In over-nourished populations, it could be beneficial to retain the phenolic compounds released into the soaking water during the cooking process as a strategy to preserve health promoter compounds. Such information should be passed to consumers and food industry.

This study showed that TPC, TFC, TPAC, and the in vitro antioxidant activity (ORAC) were found higher in the colored varieties than in the white ones. The brown market color class varieties showed the highest TPC, TFC, and TPAC values with high variability. In opposition to the colored varieties, where soaked coats represented the richest fraction, in white varieties, the soaked cotyledons had higher contribution to the total soaked seeds’ phenolic content. Despite the phenolic compounds’ loss into the soaking water, on average, more than 50% of the phenolic content remained preserved in the soaked seeds, being distributed between the two fractions, coats and cotyledons, in the different common bean varieties.

The use of UPLC-TripleTOF-MS enabled the identification of procyanidin B5 (36), (+)-catechin-7-*O*-glucoside (17) and luteolin-3′,7-di-*O*-glucoside (39) or kaempferol-3′,7-diglucoside, for the first time, in common beans. Several *p*-coumaroyl, feruloyl and synapoyl aldaric acid isomers were also identified, in higher numbers than the ones described in the literature. After the soaking process in cotyledons, the phenolic acids (hydroxybenzoic and hydroxycinnamic acids), represented the predominant class, 64.0% of the total chromatographic area. In soaking water and soaked coats, the sum of flavanols and flavonols’ classes represented, respectively, 75.9 and 93.7% of the total areas. 

Information about abundance and distribution of phenolic compounds in common bean seeds and fractions represents an affordable approach, especially when commercial standards are not available. Such compounds may be isolated from those fractions, in order to clarify their individual effect or synergistic impact in in vitro and in vivo studies of disease models.

In summary, the present study showed for the first time that the Portuguese common bean genetic resources have high diversity in the phenolic composition and associated antioxidant activity, demonstrating high potential for quality improvement through cross/selection-breeding. Additionally, the identification of bioactive phenolic compounds (e.g., procyanidins), in soaking water and soaked coats, with recognized value as prebiotic compounds in feed and food industry, and health-promoting agents, especially in communities with high prevalence of NCD (e.g., obesity or hypercholesterolemia) supports an active discussion on food preparation techniques, such as discarding the soaking water or removing the beans’ coats, for the sake of preserving the health properties of beans-based food products. In order to provide complementary information about the relevance of common beans’ phenolic compounds in human diet, more work on common beans phenolic compounds’ bioaccessibility and bioavailability should be performed.

## Figures and Tables

**Table 1:** Accession numbers (PRT 005 germplasm bank collection) of Portuguese common bean’s varieties, corresponding locations (latitude and longitude), seed color and pattern.

Sample	PRT 005 Accession nº	Latitude	Longitude	Color	Pattern
1	4144	40°51′0″	7°29′49″	White	Plain
2	5383	41°32′43″	8°25′35″	White	Plain
3	4088	40°41′51″	8°5′4″	White	Plain
4	1979	40°45′3″	7°32′14″	White	Plain
5	5249	40°39′24″	7°54′52″	White	Plain
6	5388	40°39′24″	7°54′52″	Yellow	Plain
7	4185	40°20′7″	7°9′48″	White background with brown speckles and brown marginal pattern	Speckled with marginal color
8	4189	40°20′7″	7°49′48″	White and brown backgrounds	Spotted bicolor
9	4110	40°37′60″	8°3′40″	Pink	Plain
10	4179	40°37′60″	7°23′34″	Pink	Plain
11	4182-P	40°39′37″	7°24′38″	Pink background with brown stripes	Stripes
12	4119	40°39′24″	7°54′52″	Pink background with purple stripes	Stripes
13	4097	40°41′51″	8°5′4″	Pink background with purple stripes	Stripes
14	4038	40°53′43″	7°44′49″	Pink background with purple stripes	Stripes
15	4051	40°52′50″	7°48′16″	Pink background with purple stripes	Stripes
16	5389	40°39′24″	7°54′52″	Pink background with purple stripes	Stripes
17	4120	40°39′24″	7°54′52″	Red	Plain
18	5387	40°32′19″	7°16′3″	Red	Plain
19	4070	40°54′49″	7°58′32″	Red	Plain
20	5382	41°32′43″	8°25′35″	Red	Plain
21	4149-R	40°19’33″	7°41′16″	Red	Plain
22	4081	41°11′50″	7°49′33″	Light Brown	Plain
23	4182-B	40°39′37″	7°24′38″	Brown	Plain
24	GC-34	40°32′19″	7°16′3″	Brown	Plain
25	GC-35	40°32′19″	7°16′3″	Brown	Plain
26	GC-17	40°12′7″	8°26′48″	Brown	Plain
27	4194	40°20′50″	7°51′26″	Brown background with dark brown stripes	Stripes
28	GC-40	40°32′19″	7°16′3″	Brown background with dark brown stripes	Stripes
29	5384	39°14′12″	8°41′9″	Brown background with dark brown stripes	Stripes
30	4085	40°41′51″	8°5′4″	Brown background with dark brown stripes	Stripes
31	4071	40°54′49″	7°58′32″	Brown background with dark brown stripes	Stripes

**Table 2:** Summary of the results, expressed as the average ± standard deviation (SD), obtained for the spectrophotometric parameters, TPC (Total Phenolic Content), TFC (Total Flavonoid Content), TPAC (Total Proanthocyanidin Content) and ORAC (Oxygen Radical Absorbance Capacity) in the different common bean fractions (WF—whole flour, SW—soaking water, coats and cotyledons). Yellow sample was excluded since only one sample could be classified in such color class.

Parameter	Analyzed Fraction	White (*n* = 5)	White and Brown (*n* = 2)	Pink (*n* = 8)	Red (*n* = 5)	Brown (*n* = 10)	Described in Literature
**TPC (mg GAE/g DW)**	**WF (raw)**	1.38 ± 0.22 **(a)**	2.73 ± 0.23 **(ab)**	4.58 ± 0.67 **(bc)**	4.62 ± 0.70 **(bc)**	5.12 ± 1.22 **(c)**	1.59 ± 0.08 (navy [34]); 0.37 ± 0.026 (white [35]); 0.45 ± 0.23 (navy [36]); 3.11 ± 0.14 (dark red kidney [34]); 1.24 ± 0.043 (red [35]); 2.15 ± 0.94 (red kidney [36]); 2.25 ± 0.05 (red kidney [37]); 0.60 ± 0.038 (Brown [35])
	**SW**	0.12 ± 0.05 **(a)**	1.20 ± 0.14 **(ab)**	1.98 ± 0.77 **(b)**	1.92 ± 0.66 **(b)**	2.18 ± 0.78 **(c)**	n.d
	**Coats**	0.04 ± 0.00 **(a)**	2.38 ± 0.82 **(ab)**	3.42 ± 0.93 **(b)**	3.77 ± 0.31 **(b)**	3.71 ± 0.62 **(b)**	1.20 ± 0.02 (navy); 1.16 ± 0.01 (great northern); 1.88 ± 0.00 (pink); 1.44 ± 0.00 (dark red kidney); 5.53 ± 0.87 (light red kidney); 3.79 ± 0.04 (small red) [38]
	**Cotyledons**	0.87 ± 0.06 **(a)**	1.01 ± 0.14 **(a)**	1.02 ± 0.09 **(a)**	0.96 ± 0.11 **(a)**	1.06 ± 0.16 **(a)**	2.00 ± 0.01 (navy); 1.86 ± 0.04 (great northern); 1.97 ± 0.01 (pink); 2.11 ± 0.06 (dark red kidney); 2.15 ± 0.04 (light red kidney); 2.02 ± 0.08 (small red) [38]
**TFC (mg CE/g DW)**	**WF (raw)**	0.14 ± 0.04 **(a)**	0.77 ± 0.00 **(b)**	2.01 ± 0.70 **(c)**	1.45 ± 0.24 **(c)**	1.85 ± 0.64 **(c)**	0.85 ± 0.03 (red kidney [37])
	**SW**	0.01 ± 0.00 **(a)**	0.59 ± 0.35 **(ab)**	1.33 ± 0.49 **(b)**	1.13 ± 0.22 **(b)**	1.33 ± 0.55 **(b)**	n.d
	**Coats**	0.01 ± 0.00 **(a)**	1.67 ± 0.61 **(ab)**	2.26 ± 0.68 **(b)**	2.36 ± 0.13 **(b)**	2.31 ± 0.38 **(b)**	n.d
	**Cotyledons**	0.15 ± 0.01 **(a)**	0.17 ± 0.01 **(ab)**	0.21 ± 0.03 **(bc)**	0.23 ± 0.04 **(bc)**	0.22 ± 0.04 **(c)**	n.d
**TPAC (mg CE/g DW)**	**WF (raw)**	0.02 ± 0.01 **(a)**	0.29 ± 0.25 **(ab)**	0.70 ± 0.49 **(b)**	0.65 ± 0.37 **(b)**	0.65 ± 0.39 **(b)**	0.30 ± 0.03 (red kidney [37])
	**SW**	0.04 ± 0.01 **(a)**	0.07 ± 0.03 **(ab)**	0.62 ± 0.35 **(c)**	0.46 ± 0.36 **(bc)**	0.38 ± 0.27 **(bc)**	n.d
	**Coats**	0.01 ± 0.00 **(a)**	1.24 ± 0.21 **(ab)**	2.49 ± 1.02 **(bc)**	2.38 ± 0.70 **(bc)**	2.60 ± 0.75 **(c)**	n.d
**ORAC (μmol TEAC/ g DW)**	**WF (raw)**	37.35 ± 4.77 **(a)**	76.00 ± 2.59 **(b)**	143.22 ± 35.39 **(c)**	125.46 ± 31.50 **(bc)**	154.83 ± 40.41 **(c)**	59.41 ± 3.26 (red kidney [37])

Equal letters per parameter (row) indicate absence of significant differences between color classes (*p* > 0.05); n.d. not described.

**Table 3:** Phenolic compounds identified in sample’s 22 fractions.

						Soaking Water			Soaked Coats			Soaked Cotyledons			Whole Flour			References
Class	Compound	CF	Expected Mass Da	Fragments MS^2^	RT Average ± SD (min)	Found at Mass Da [M-H]-	Error ppm	IS	Found at Mass Da [M-H]-	Error ppm	IS	Found at Mass Da [M-H]-	Error ppm	IS	Found at Mass Da [M-H]-	Error ppm	IS	
**HBA**	(**1**) Protocatechuic acid-4-*O*-Gl	C_13_H_16_O_9_	316.0794	108.0241/109.0319/152.0150/153.0229	27.8 ± 0.1	315.0722	0.0	0.8835	315.0724	0.8	0.8915	315.0716	−1.8	0.8896	315.0701	−6.5	0.7409	[49]
	(**2**) Vanillic acid	C_8_H_8_O_4_	168.0423	108.0240/123.0489/124.0157/152.0158	28.1 ± 0.1	167.0351	1.0	0.9916	167.0352	1.4	0.7308	167.0351	0.7	0.9901	167.0351	0.8	0.9683	[50,51]
	(**5**) Protocatechuic acid*	C_7_H_6_O_4_	154.0266	108.0241/109.0325	31.4 ± 0.1	153.0196	1.7	0.9835	153.0200	4.4	0.9558	153.0195	0.9	0.9721	153.0194	0.5	0.9770	[52,53]
	(**12**) *p*-Hydroxybenzoic acid-4-*O*-Gl	C_13_H_16_O_8_	300.0845	93.0372/137.0304	36.5 ± 0.1	299.0775	0.9	0.8742	299.0770	−0.8	0.8896	299.0771	−0.5	0.8573	299.0756	−5.6	0.9116	[49]
	(**25**) *p*-Hydroxybenzoic acid*	C_7_H_6_O_3_	138.0317	65.0413/93.0369	41.4 ± 0.1	137.0246	1.0	0.9898	137.0245	0.7	0.9804	137.0246	1.0	0.9797	137.0245	0.7	0.9880	[51]
	(**27**) Gentisic acid*	C_7_H_6_O_4_	154.0266	108.0241/109.0322	43.4 ± 0.1	153.0199	3.8	0.9684	153.0195	1.2	0.9061	153.0194	0.6	0.9798	153.0193	−0.4	0.8411	[13,54]
**HCA**	(**3**) *p*-Coumaroyl aldaric acid 1	C_15_H_16_O_10_	356.0743	57.0358/59.0152/85.0312/89.0261/119.0370/129.0215/147.0313/191.0233/209.0339	29.5 ± 0.1	355.0673	0.6	0.9334	355.0653	−4.9	0.9543	355.0745	20.8	0.9266	355.0714	12.3	0.9840	[51]
	(**6**) *p*-Coumaroyl aldaric acid 2	C_15_H_16_O_10_	356.0743	57.0363/59.0156/85.0316/129.0216/147.0330/163.0428/191.0235/209.0343	32.7 ± 0.1	355.067	−0.3	0.8553	355.0656	−4.2	0.9724	355.0657	−3.9	0.8463	355.0654	−4.6	0.8660	[51]
	(**7**) Feruloyl aldaric acid 1	C_16_H_18_O_11_	386.0849	57.0362/59.0154/85.0315/129.0216/147.0327/191.0234/209.0339	33.4 ± 0.1	385.0772	−1.1	0.8642	385.0755	−5.5	0.9330	385.0761	−4.0	0.8615	385.0761	−3.9	0.8802	[51,55]
	(**8**) *p*-Coumaroyl aldaric acid 3	C_15_H_16_O_10_	356.0743	57.0364/59.0159/85.0320/129.0225/147.0336/ 191.0246/ 209.0357	34.9 ± 0.1	355.0672	0.3	0.8547	355.0659	−3.4	0.9032	355.0659	−3.3	0.8706	355.0655	−4.4	0.8581	ND
**HCA**	(**9**) Sinapoyl aldaric acid 1	C_17_H_20_O_12_	416.0955	57.0363/59.0155/85.0316/129.0220/147.0329/191.0230/209.0338	35.0 ± 0.1	415.0869	−3.0	0.9592	415.0878	−1.0	0.9388	415.0882	0.1	0.9354	415.0856	−6.4	0.8778	[55]
	(**10**) Feruloyl aldaric acid 2	C_16_H_18_O_11_	386.0849	57.0362/85.0312/147.0323/191.0227/209.0339/223.0495	35.3 ± 0.1	385.0761	−4.0	0.9050	385.0755	−5.4	0.9710	385.0761	−3.9	0.8701	385.0756	−5.3	0.8887	[51,55]
	(**13**) Feruloyl aldaric acid 3	C_16_H_18_O_11_	386.0849	57.0359/59.0154/85.0311/129.0217/147.0319/191.0228/193.0539/209.0331	36.6 ± 0.1	385.0764	−3.3	0.9242	385.0753	−6.0	0.9524	385.0763	−3.4	0.8808	385.0758	−4.8	0.8981	[51,55]
	(**14**) *p*-Coumaroyl aldaric acid 4	C_15_H_16_O_10_	356.0743	85.0315/147.0326/191.0233/209.0343	36.8 ± 0.2	355.0665	−1.6	0.8922	355.0653	−4.8	0.9581	355.0659	−3.2	0.8631	355.065	−5.7	0.8873	ND
	(**15**) *p*-Coumaroyl aldaric acid 5	C_15_H_16_O_10_	356.0743	57.0361/59.0153/85.0315/129.0216/147.0324/191.0234/209.0343	37.6 ± 0.1	355.0664	−1.9	0.8651	355.0658	−3.6	0.9319	355.0660	−3.1	0.8480	355.0655	−4.5	0.8628	ND
	(**16**) Feruloyl aldaric acid 4	C_16_H_18_O_11_	386.0849	57.0361/85.0313/129.0211/147.0322/191.0228/209.0333	38.2 ± 0.1	385.0775	−0.5	0.8943	385.0762	−3.7	0.9035	385.0766	−2.6	0.8748	385.076	−4.3	0.8851	[55]
	(**19**) Sinapoyl aldaric acid 2	C_17_H_20_O_12_	416.0955	57.0360/59.0154/85.0314/129.0214/147.0327/191.0234/209.0339/223.0661	38.7 ± 0.1	415.0865	−4.0	0.9481	415.0856	−6.2	0.9243	415.0855	−6.4	0.9349	415.0851	−7.5	0.9162	[55]
	(**20**) Sinapoyl aldaric acid 3	C_17_H_20_O_12_	416.0955	57.0363/85.0317/129.0220/147.0329/191.0237/209.0343	39.4 ± 0.1	415.0871	−2.5	0.8585	415.0855	−6.5	0.9589	415.0859	−5.5	0.8748	415.0857	−6.1	0.8622	ND
	(**22**) Feruloyl aldaric acid 5	C_16_H_18_O_11_	386.0849	57.0363/85.0315/129.0216/147.0326/191.0232/209.0342	40.2 ± 0.1	385.0774	−0.7	0.8745	385.0758	−4.8	0.9144	385.0767	−2.5	0.8842	385.0762	−3.6	0.8726	[55]
	(**23**) Sinapoyl aldaric acid 4	C_17_H_20_O_12_	416.0955	57.0362/59.0154/85.0315/129.0215/147.0324/191.0234/209.0343	40.8 ± 0.1	415.0861	−5.0	0.9706	415.0853	−7.1	0.9082	415.0861	−4.9	0.8771	415.0856	−6.3	0.8977	ND
**HCA**	(**26**) *p*-Coumaroyl aldaric acid 6	C_15_H_16_O_10_	356.0743	57.0361/59.0154/85.0313/ 129.0212/147.0326/163.0420/191.0232/209.0339	41.8 ± 0.1	NF	-	-	355.0654	−4.7	0.7331	355.0658	−3.6	0.8664	355.0654	−4.7	0.8717	ND
	(**30**) Feruloyl aldaric acid 6	C_16_H_18_O_11_	386.0849	57.0364/59.0157/85.0317/111.0106/129.0218/147.0333/191.0241/209.0348	44.5 ± 0.1	385.0772	−1.0	0.8900	385.0760	−4.1	0.8934	385.0766	−2.8	0.9271	385.0764	−3.2	0.8801	ND
	(**31**) Sinapoyl aldaric acid 5	C_17_H_20_O_12_	416.0955	57.0361/59.0152/85.0314/129.0219/147.0326/191.0232/209.0339	44.8 ± 0.1	415.0871	−2.7	0.8610	415.0857	−6.1	0.9493	415.0863	−4.7	0.8794	415.0858	−5.9	0.8865	ND
	(**40**) *p*-Coumaric acid*	C_9_H_8_O_3_	164.0473	93.0364/117.0369/119.0529	58.9 ± 0.2	163.0407	4.2	0.9776	163.0404	1.9	0.9503	NF	-	-	163.0401	0.2	0.8870	[25]
	(**43**) Sinapic acid*	C_11_H_12_O_5_	224.0685	93.0361/121.0308/149.0272/163.0423/164.05077/165.0227/193.0175/208.0417	63.1 ± 0.2	223.0613	0.6	0.9604	223.0610	−1.0	0.7187	223.0611	−0.5	0.9814	223.0605	−3.1	0.9721	[25]
	(**44**) Ferulic acid*	C_10_H_10_O_4_	194.0579	102.9351/133.0316/134.0400/149.0636/178.0305	63.8 ± 0.2	193.0510	1.9	0.9545	193.0506	−0.3	0.8344	NF	-	-	193.0507	0.3	0.9355	[25,51]
**Flavanol**	(**4**) (+)-Catechin 3′-*O*-glucose	C_21_H_24_O_11_	452.1319	137.0279/245.0874/289.0836/299.0835	30.8 ± 0.1	451.1234	−2.5	0.8674	451.1227	−4.2	0.8103	451.1220	−5.8	0.8440	451.1218	−6.1	0.8412	[51]
	(**11**) Procyanidin B1	C_30_H_26_O_12_	578.1424	125.0272/161.0276/287.0613/289.0771/407.0836/425.0935/451.1095	36.3 ± 0.1	577.1338	−2.4	0.8707	577.1322	−5.1	0.8217	NF	-	-	577.1308	−7.6	0.8528	[51]
	(**17**) (+)-Catechin 7-*O*-*β*-D-Gl	C_21_H_24_O_11_	452.1319	245.0860/289.0766	37.7 ± 0.1	451.124	−1.3	0.8408	451.1226	−4.5	0.8522	451.1216	−6.6	0.9377	451.1214	−7.1	0.8410	[56]
	(**18**) Procyanidin B2*	C_30_H_26_O_12_	578.1424	125.0270/289.0768/407.0831/425.0936/451.1091	38.3 ± 0.1	577.1334	−3.0	0.8429	577.1320	−5.5	0.7773	577.1306	−7.8	0.7106	577.1306	−7.8	0.8349	[51]
	(**21**) Procyanidin C1	C_45_H_38_O_18_	866.2058	575.1276/577.1431/695.1510/713.1622	40.2 ± 0.1	865.1957	−3.3	0.7994	865.1925	−7.0	0.7427	865.1950	−4.1	0.5898	865.1907	−9.0	0.8559	[43,57]
**Flavanol**	(**24**) *(+)*-Catechin*	C_15_H_14_O_6_	290.079	109.0321/123.0479/125.0272/137.0274/151.0433/179.0388/203.0753/205.0546/221.0865/245.0866	40.6 ± 0.1	289.0719	0.4	0.8594	289.0718	0.0	0.8649	289.0711	−2.1	0.9019	289.0714	−1.1	0.8714	[51]
	(**28**) Procyanidin B3	C_30_H_26_O_12_	578.1424	125.0267/287.0609/289.0762/407.0829/425.0932/451.1094	43.7 ± 0.1	577.1331	−3.5	0.8371	577.1312	−6.9	0.8396	NF	-	-	577.1290	−10.7	0.9183	[51]
	(**32**) Procyanidin B4	C_30_H_26_O_12_	578.1424	125.0266/161.0267/287.0598/289.0756/407.0813/425.0923/451.1092	46.4 ± 0.1	577.1327	−4.2	0.8305	577.1310	−7.2	0.8814	NF	-	-	577.1294	−9.9	0.7277	[53]
	(**33**) (-)-Epicatechin*	C_15_H_14_O_6_	290.079	109.0317/123.0476/125.0267/151.0425/203.0749/205.0537/245.0860	46.8 ± 0.1	289.0719	0.6	0.8671	289.0716	−0.4	0.8691	289.0705	−4.2	0.9701	289.0712	−1.8	0.8682	[58]
	(**35**) Procyanidin C2	C_45_H_38_O_18_	866.2058	575.1269	48.1 ± 0.2	865.1952	−3.9	0.7947	865.1920	−7.6	0.7222	865.1909	−8.8	0.8242	865.1908	−8.9	0.7730	[57]
	(**36**) Procyanidin B5	C_30_H_26_O_12_	578.1424	125.0266/287.0603/289.0760/407.0825/425.0924/451.1082	50.8 ± 0.2	577.1334	−3.0	0.8527	577.1316	−6.2	0.8447	577.1296	−9.6	0.7375	577.1308	−7.6	0.8452	[59]
**Flavanones**	(**29**) Eriodictyol-hexoside 1	C_21_H_22_O_11_	450.1162	125.0267/179.0017/243.0698/259.0662/283.0657/287.0615/301.0764/421.1202	43.7 ± 0.1	449.1074	−3.4	0.8475	449.1054	−8.0	0.8682	449.1052	−8.3	0.8673	449.1052	−8.4	0.8317	[51,55]
	(**34**) Eriodictyol-hexoside 2	C_21_H_22_O_11_	450.1162	125.0273/259.0672/269.0510/287.0618	47.2 ± 0.1	449.108	−2.0	0.8735	449.1068	−4.6	0.8607	449.1070	−4.3	0.8465	449.1061	−6.2	0.8569	[55]
	(**37**) Eriodictyol-hexoside 3	C_21_H_22_O_11_	450.1162	259.0651/287.0607	53.6 ± 0.1	449.1077	−2.7	0.8574	449.1060	−6.6	0.9051	449.1057	−7.3	0.8843	449.1064	−5.7	0.8703	ND
**Flavanones**	(**38**) Eriodictyol-hexoside 4	C_21_H_22_O_11_	450.1162	125.0262/151.0061/152.0143/179.0014/180.0094/269.0497/287.0590	56.7 ± 0.2	449.1078	−2.6	0.8554	449.1056	−7.5	0.9268	449.1056	−7.4	0.9693	449.1062	−6.1	0.8633	ND
	(**42**) Naringenin-7-Gl	C_21_H_22_O_10_	434.1213	119.0521/151.0057/271.0647/313.0589	60.4 ± 0.2	433.1119	−4.8	0.9503	433.1107	−7.6	0.8883	433.1088	−12.0	0.7686	433.1097	−10.0	0.9045	[53]
	(**50**) Eriodictyol	C_15_H_12_O_6_	288.0634	125.0269/243.0703/259.0659	82.7 ± 0.2	287.0561	−0.1	0.9315	287.0557	−1.5	0.8979	287.0547	−4.8	0.9471	287.0553	−2.7	0.9044	[51]
	(**53**) Naringenin	C_15_H_12_O_5_	272.0685	107.0160/119.0524/151.0058/177.0228	95.4 ± 0.1	271.0610	−0.7	0.9873	271.0605	−2.6	0.9748	271.0605	-2.7	0.9126	271.0602	−3.8	0.9438	[51]
**Flavonols**	(**39**) Luteolin 3′,7-di-*O*-Gl or kaempferol-3′,7-dihexoside	C_27_H_30_O_16_	610.1534	284.0373/285.0446	56.8 ± 0.2	609.1442	−3.1	0.8322	609.1415	−7.5	0.8720	609.1409	−8.6	0.9374	609.1417	−7.3	0.8715	[60]
	(**41**) Rutin*	C_27_H_30_O_16_	610.1534	284.0370/285.0453/300.0320/301.0386/327.0554/607.2499	59.9 ± 0.2	609.1441	−3.2	0.8302	609.1421	−6.6	0.8892	609.1418	−7.1	0.8585	609.1410	−8.3	0.8494	[25,55]
	(**45**) Kaempferol-3-*O*-xylosyl-Gl	C_26_H_28_O_15_	580.1428	284.0377/285.0453/429.0891	64.7 ± 0.1	579.1335	−3.5	0.8194	NF	-	-	NF	-	-	579.1316	−6.9	0.8276	[25,61]
	(**46**) Quercetin-3-*O*-Gl	C_21_H_20_O_12_	464.0955	300.0318/301.0392	65.0 ± 0.2	463.0867	−3.2	0.8117	463.0855	−5.9	0.8163	NF	-	-	463.0849	−7.1	0.8265	[25,61]
	(**47**) Quercetin-3-(6-*O*-acetyl-*β*-Gl)	C_23_H_22_O_13_	506.106	300.0319/301.0399/463.0923	73.2 ± 0.2	505.0970	−3.5	0.8666	505.0942	−9.1	0.9576	NF	-	-	505.0952	−7.0	0.8700	[55,62]
	(**48**) Kaempferol-3-*O*-Gl	C_21_H_20_O_11_	448.1006	227.0397/255.0363/284.0447/285.0476	78.1 ± 0.1	447.0934	0.3	0.8361	447.0918	−3.4	0.8064	447.0904	−6.4	0.8319	447.0909	−5.2	0.8716	[25,55,61]
	(**49**) Kaempferol-3-*O*-acetyl-Gl	C_23_H_22_O_12_	490.1111	255.0339/284.0386/285.0451	82.4 ± 0.1	489.1027	−2.2	0.8634	489.1004	−7.1	0.8248	489.1006	−6.5	0.8577	489.1010	−5.8	0.8644	[61]
	(**52**) Quercetin*	C_15_H_10_O_7_	302.0427	107.0143/121.0312/151.0059/179.0017/255.2372/273.0434	91.2 ± 0.1	301.0347	−2.1	0.9273	301.0353	−0.2	0.8619	301.0341	−4.3	0.9211	301.0336	−5.9	0.9445	[25,51]
**Flavonols**	(**55**) Kaempferol*	C_15_H_10_O_6_	286.0477	93.0368/107.0159/151.0062/159.0479/185.0639/187.0428/211.0429/229.0545/239.0385/257.0493	97.4 ± 0.1	285.0407	1.0	0.8786	285.0403	−0.5	0.8557	285.0402	−1.0	0.8565	285.0400	−1.6	0.8669	[25,61]
**Isoflavones**	(**51**) Daidzein*	C_15_H_10_O_4_	254.0579	209.0643/224.0538/225.0617	88.5 ± 0.1	253.0505	−0.4	0.9479	253.0503	−1.2	0.8295	253.0502	−1.7	0.9510	253.0495	−4.4	0.7987	[61]
	(**54**) Genistein*	C_15_H_10_O_5_	270.0528	133.0309	96.3 ± 0.1	269.0452	−1.2	0.7351	269.0447	−3.1	0.9337	269.0449	−2.5	0.8803	NF	-	-	[61]

CF—Chemical Formula; IS—Isotope Score; RT—Retention Time; SD—Standard Deviation; HBA—Hydroxybenzoic Acid; HCA—Hydroxycinnamic Acid; Gl—Glucoside; * Compounds identified by comparison with standards; the major fragments are underlined; NF—Not Found; ND—Not Described.

**Table 4:** Relative quantification of identified compounds in sample’s 22 fractions.

		Soaking Water		Soaked Coats		Soaked Cotyledons		Whole Flour (Raw)	
Class	Name	Area (%, Area/Total Area)	%, Area/Total Compounds’ Class Area	Area (%, Area/Total Area)	%, Area/Total Compounds’ Class Area	Area (%, Area/Total Area)	%, Area/Total Compounds’ Class Area	Area (%, Area/Total Area)	%, Area/Total Compounds’ Class Area
**HBA**	(**1**) Protocatechuic acid-4-*O*-Gl	1677120 (2.9)	43.2	85308 (0.6)	20.5	451623 (4.8)	31.2	13153 (0.03)	8.1
	(**2**) Vanillic acid	67863 (0.1)	1.7	4363 (0.03)	1.0	15603 (0.2)	1.1	29105 (0.1)	17.9
	(**5**) Protocatechuic acid	130651 (0.2)	3.4	137055 (1.0)	33.0	9253 (0.1)	0.6	25185 (0.1)	15.5
	(**12**) *p*-Hydroxybenzoic acid-4-*O*- Gl	1841267 (3.2)	47.4	163157 (1.2)	39.3	949047 (10.1)	65.6	23743 (0.1)	14.6
	(**25**) *p*-Hydroxybenzoic acid	60267 (0.1)	1.6	19416 (0.1)	4.7	13706 (0.1)	0.9	69860 (0.2)	43.0
	(**27**) Gentisic acid	106891 (0.2)	2.8	6345 (0.05)	1.5	7149 (0.1)	0.5	1446 (0.003)	0.9
	**Total compounds’ class area**	3884059 (6.6)	100.0	415644 (3.0)	100.0	1446381 (14.7)	100.0	162493 (0.4)	100.0
**HCA**	(**3**) *p*-Coumaroyl aldaric acid 1	26590 (0.05)	0.6	4318 (0.03)	2.2	167283 (1.8)	3.8	114749 (0.3)	1.5
	(**6**) *p*-Coumaroyl aldaric acid 2	139230 (0.2)	3.3	8798 (0.1)	4.5	42992 (0.5)	1.0	73863 (0.2)	1.0
	(**7**) Feruloyl aldaric acid 1	97092 (0.2)	2.3	7766 (0.1)	4.0	305601 (3.3)	6.9	689964 (1.7)	9.2
	(**8**) *p*-Coumaroyl aldaric acid 3	1265546 (2.2)	30.4	22910 (0.2)	11.7	417109 (4.4)	9.5	496423 (1.2)	6.6
	(**9**) Sinapoyl aldaric acid 1	35954 (0.1)	0.9	1977 (0.01)	1.0	65773 (0.7)	1.5	183550 (0.4)	2.5
	(**10**) Feruloyl aldaric acid 2	12142 (0.02)	0.3	3078 (0.02)	1.6	137695 (1.5)	3.1	130905 (0.3)	1.7
	(**13**) Feruloyl aldaric acid 3	66532 (0.1)	1.6	3551 (0.03)	1.8	49572 (0.5)	1.1	139151 (0.3)	1.9
	(**14**) *p*-Coumaroyl aldaric acid 4	321109 (0.6)	7.7	5263 (0.04)	2.7	9651 (0.1)	0.2	9516 (0.02)	0.1
	(**15**) *p*-Coumaroyl aldaric acid 5	150575 (0.3)	3.6	17330 (0.1)	8.9	396951 (4.2)	9.0	339760 (0.8)	4.5
	(**16**) Feruloyl aldaric acid 4	139602 (0.2)	3.3	3934 (0.03)	2.0	213762 (2.3)	4.9	125167 (0.3)	1.7
	(**19**) Sinapoyl aldaric acid 2	49814 (0.1)	1.2	1705 (0.01)	0.9	55150 (0.6)	1.3	14544 (0.03)	0.2
	(**20**) Sinapoyl aldaric acid 3	172631 (0.3)	4.1	5470 (0.04)	2.8	242307 (2.6)	5.5	677720 (1.6)	9.0
	(**22**) Feruloyl aldaric acid 5	470732 (0.8)	11.3	44560 (0.3)	22.8	757939 (8.1)	17.2	1381715 (3.3)	18.4
	(**23**) Sinapoyl aldaric acid 4	171674 (0.3)	4.1	8603 (0.06)	4.4	291186 (3.1)	6.6	747370 (1.8)	10.0
	(**26**) *p*-Coumaroyl aldaric acid 6	NF	NF	18685 (0.1)	9.6	433166 (4.4)	9.0	213995 (0.5)	2.9
	(**30**) Feruloyl aldaric acid 6	730915 (1.3)	17.5	28548 (0.2)	14.6	971927 (10.4)	22.1	1644680 (4.0)	22.0
	(**31**) Sinapoyl aldaric acid 5	185506 (0.3)	4.5	7616 (0.05)	3.9	273562 (2.9)	6.2	465873 (1.1)	6.2
	(**40**) *p*-Coumaric acid	89291 (0.2)	2.1	338 (0.002)	0.2	NF	NF	11431 (0.03)	0.2
	(**43**) Sinapic acid	2151 (0.004)	0.1	418 (0.003)	0.2	8055 (0.1)	0.2	6305 (0.02)	0.1
	(**44**) Ferulic acid	40918 (0.1)	1.0	158 (0.001)	0.1	NF	NF	23022 (0.1)	0.3
	**Total compounds’ class area**	4168002 (7.1)	100.0	195028 (1.4)	100.0	4839680 (49.3)	100.0	7489702 (18.0)	100.0
**Flavanols**	(**4**) (+)-Catechin 3’-*O*-Gl	7636898 (13.0)	33.5	1165545 (8.3)	32.7	518148 (5.3)	86.3	3166476 (7.6)	31.6
	(**11**) Procyanidin B1	4199734 (7.2)	18.4	866524 (6.2)	24.3	NF	NF	1885313 (4.5)	18.8
	(**17**) (+)-Catechin 7-*O*-Gl	1214896 (2.1)	5.3	70095 (0.5)	2.0	10469 (0.1)	1.7	172724 (0.4)	1.7
	(**18**) Procyanidin B2	926982 (1.6)	4.1	197566 (1.4)	5.5	2303 (0.02)	0.4	468641 (1.1)	4.7
	(**21**) Procyanidin C1	1216935 (2.1)	5.3	546543 (3.9)	15.3	2018 (0.02)	0.3	265008 (0.6)	2.6
	(**24**) (+)-Catechin	5800883 (9.9)	25.4	507760 (3.6)	14.2	60537 (0.6)	10.1	3025744 (7.3)	30.2
	(**28**) Procyanidin B3	205083 (0.4)	0.9	21266 (0.2)	0.6	NF	NF	38912 (0.1)	0.4
	(**32**) Procyanidin B4	60776 (0.1)	0.3	8658 (0.1)	0.2	NF	NF	28180 (0.1)	0.3
	(**33**) (-)-Epicatechin	559522 (1.0)	2.5	51252 (0.4)	1.4	4432 (0.05)	0.7	353630 (0.8)	3.5
	(**35**) Procyanidin C2	185749 (0.3)	0.8	25218 (0.2)	0.7	171 (0.002)	0.0	85610 (0.2)	0.9
	(**36**) Procyanidin B5	820786 (1.4)	3.6	104129 (0.7)	2.9	2311 (0.02)	0.4	522375 (1.3)	5.2
	**Total compounds’ class area**	22828243 (39.0)	100.0	3564555 (25.5)	100.0	600389 (6.1)	100.0	10012612 (24.0)	100.0
**Flavanones**	(**29**) Eriodictyol-hexoside 1	98920 (0.2)	1.7	4350 (0.03)	1.7	4011 (0.04)	1.3	197075 (0.5)	2.7
	(**34**) Eriodictyol-hexoside 2	5166817 (8.8)	86.4	178852 (1.3)	70.6	272049 (2.8)	90.9	5800730 (13.9)	80.6
	(**37**) Eriodictyol-hexoside 3	252875 (0.4)	4.2	25333 (0.2)	10.0	12016 (0.1)	4.0	844769 (2.0)	11.7
	(**38**) Eriodictyol-hexoside 4	177730 (0.3)	3.0	5960 (0.04)	2.4	804 (0.01)	0.3	165914 (0.4)	2.3
	(**42**) Naringenin-7-Gl	10713 (0.02)	0.2	585 (0.004)	0.2	409 (0.004)	0.1	5652 (0.01)	0.1
	(**50**) Eriodictyol	254008 (0.4)	4.2	28839 (0.2)	11.4	1324 (0.01)	0.4	161919 (0.4)	2.3
	(**53**) Naringenin	15799 (0.03)	0.3	9365 (0.1)	3.7	8739 (0.1)	2.9	17103 (0.04)	0.2
	**Total compounds’ class area**	5976862 (10.2)	100.0	253284 (1.8)	100.0	299352 (3.0)	100.0	7193164 (17.3)	100.0
**Flavonols**	(**39**) Luteolin 3,7-di-*O*-Gl or Kaempferol-3′,7-dihexoside	309103 (0.5)	1.4	20371 (0.1)	0.2	2187 (0.02)	0.1	535830 (1.3)	3.2
	(**41**) Rutin	208461 (0.4)	1.0	20628 (0.1)	0.2	14381 (0.1)	0.6	86221 (0.2)	0.5
	(**45**) Kaempferol-3-*O*-xylosyl-Gl	2737477 (4.7)	12.7	NF	NF	NF	NF	2217478 (5.3)	13.2
	(**46**) Quercetin-3-*O*-Gl	1233782 (2.1)	5.7	8850 (0.1)	0.1	NF	NF	1485967 (3.6)	8.9
	(**47**) Quercetin-3-(6-*O*-acetyl-Gl)	62179 (0.1)	0.3	37 (0.003)	0.0	NF	NF	63797 (0.2)	0.4
	(**48**) Kaempferol-3-*O*-Gl	11475034 (19.6)	53.2	6344589 (45.4)	66.5	438101 (4.5)	16.8	7437720 (17.9)	44.3
	(**49**) Kaempferol-3-*O*-acetyl-Gl	5393293 (9.2)	25.0	1228219 (8.8)	12.9	1942204 (19.8)	74.7	4250372 (10.2)	25.3
	(**52**) Quercetin	8100 (0.01)	0.0	12113 (0.1)	0.1	10403 (0.1)	0.4	17618 (0.04)	0.1
**Flavonols**	(**55**) Kaempferol	145232 (0.3)	0.7	1903123 (13.6)	20.0	194088 (2.0)	7.5	682007 (1.6)	4.1
	**Total compounds’ class area**	21572661 (36.9)	100.0	9537931 (68.2)	100.0	2601364 (27.7)	100.0	16777009 (40.3)	100.0
**Isoflavones**	(**51**) Daidzein	2243 (0.004)	25.2	2434 (0.02)	23.0	9867 (0.1)	34.6	538 (0.001)	100.0
	(**54**) Genistein	6664 (0.01)	74.8	8165 (0.1)	77.0	18661 (0.2)	65.4	NF	NF
	**Total compounds’ class area**	8907 (0.02)	100.0	10599 (0.1)	100.0	28528 (0.3)	100.0	538 (0.001)	100.0
	**Total area**	58438734 (100.0)		13977041 (100.0)		9815694 (100.0)		41635518 (100.0)	

HBA—Hydroxybenzoic Acid; HCA—Hydroxycinnamic Acid; Gl—Glucoside; NF—Not Found; the underlined percentage(s) indicate, per compounds’ class the most abundant compound(s) in each fraction.

## Supplementary Materials

The following are available online at https://www.mdpi.com/2304-8158/8/8/296/s1, Table S1: Average values ± Standard Deviation of Total Phenolic Content (TPC), mg GAE/g DW, Total Flavonoid Content (TFC), mg CE/g DW, and Total Proanthocyanidin Content (TPAC), mg CE/g DW, determined in common bean whole flour (WF) and soaking water (SW). ORAC, µmol TEAC/g DW was determined in whole flour (WF). TPC(SW)/TPC(WF), TFC(SW)/TFC(WF), TPAC(SW)/TPAC(WF) are, respectively, the percentage of TPC, TFC and TPAC released into the soaking water, Table S2: Average values ± Standard Deviation of Total Phenolic Content (TPC), mg GAE/g DW, Total Flavonoid Content (TFC), mg CE/g DW, and Total Proanthocyanidin Content (TPAC), mg CE/g DW, determined in soaked cotyledons (Cot) and coats (C), Table S3: Contribution of the different parameters analyzed in the two first principal components (* loadings ≥ |0.400|), Table S4: Average ± standard deviation values of the parameters analyzed in the different fractions, considering the clusters of common bean varieties defined by multivariate analysis. Different letters indicate values significantly different between clusters (Scheffé’s test, *p* < 0.05), Table S5: Discriminant analysis to evaluate the clustering solution, Table S6: Molecular structure of the compounds identified.
