# Supplementary material for: Characterization of Soaking Process’ Impact in Common Beans Phenolic Composition: Contribute from the Unexplored Portuguese Germplasm
Source: Foods. 2019 Jul 28;8(8):296. doi: 10.3390/foods8080296 (PMC6723714; doi:10.3390/foods8080296)
Supplement: Supplementary file 1 [file foods-08-00296-s001.pdf]

Supplementary materials

Table S1. Average values ± Standard Deviation of Total Phenolic Content (TPC), mg GAE/g DW, Total Flavonoid Content (TFC), mg CE/g DW, and Total Proanthocyanidin Content (TPAC), mg CE/g DW, determined in common bean whole flour (WF) and soaking water (SW). ORAC, µmol TEAC/g DW, was determined in whole flour (WF).**TPC(SW)/ TPC(WF), TFC(SW)/ TFC(WF), TPAC(SW)/ TPAC(WF)** are, respectively, the percentage of TPC, TFC and TPAC released into the soaking water

Sample	PRT 005 accession nº	Moisture Content (%)	Whole Flour				Soaking Water					
			TPC_WF	TFC_WF	TPAC_WF	ORAC_WF	TPC_SW	TPC(SW)/ TPC(WF) (%)*	TFC_SW	TFC(SW)/ TFC(WF) (%)	TPAC_SW	TPAC(SW)/ TPAC(WF) (%)
**White varieties**												
**1**	**4144**	12.98	1.68 ± 0.04	0.10 ± 0.00	0.02 ± 0.00	33.31 ± 2.41	0.09 ± 0.00	5	0.01 ± 0.00	10	0.04 ± 0.01	>100
**2**	**5383**	10.25	1.12 ± 0.03	0.14 ± 0.01	0.01 ± 0.00	36.79 ± 2.96	0.13 ± 0.00	12	0.01 ± 0.00	7	0.04 ± 0.01	>100
**3**	**4088**	12.98	1.47 ± 0.01	0.18 ± 0.01	0.01 ± 0.00	44.63 ± 0.26	0.21 ± 0.01	14	0.01 ± 0.00	6	0.04 ± 0.01	>100
**4**	**1979**	10.17	1.41 ± 0.06	0.10 ± 0.01	0.02 ± 0.00	33.04 ± 1.59	0.09 ± 0.00	6	0.01 ± 0.00	10	0.04 ± 0.00	>100
**5**	**5249**	12.98	1.23 ± 0.04	0.20 ± 0.01	0.01 ± 0.00	39.00 ± 1.90	0.09 ± 0.00	7	0.01 ± 0.00	5	0.02 ± 0.00	>100
**Yellow variety**												
**6**	**5388**	12.98	3.12 ± 0.12	0.97 ± 0.04	0.15 ± 0.01	93.54 ± 1.24	0.83 ± 0.10	27	0.66 ± 0.07	68	0.11 ± 0.01	73
**White background variety with brown speckles and brown marginal pattern**												
**7**	**4185**	9.37	2.89 ± 0.03	0.78 ± 0.01	0.47 ± 0.14	74.17 ± 3.57	1.29 ± 0.07	45	0.83 ± 0.03	>100	0.09 ± 0.02	19
**White and brown background variety**												
**8**	**4189**	10.89	2.57 ± 0.17	0.77 ± 0.01	0.12 ± 0.02	77.84 ± 1.82	1.10 ± 0.05	43	0.34 ± 0.02	44	0.04 ± 0.00	33
**Pink varieties**												
**9**	**4110**	12.98	5.65 ± 0.14	2.88 ± 0.03	0.75 ± 0.05	187.72 ± 2.75	1.74 ± 0.03	31	1.03 ± 0.07	36	0.52 ± 0.13	69
**10**	**4179**	10.59	4.35 ± 0.00	1.08 ± 0.03	0.08 ± 0.00	137.46 ± 6.81	0.57 ± 0.03	13	0.35 ± 0.01	32	0.06 ± 0.01	75
**Pink background variety with brown stripes**												
**11**	**4182-P**	12.98	3.52 ± 0.09	1.38 ± 0.07	0.06 ± 0.00	93.92 ± 1.86	1.56 ± 0.05	44	0.90 ± 0.02	65	0.31 ± 0.06	>100
**Pink background varieties with purple stripes**												
**12**	**4119**	12.98	5.06 ± 0.10	2.78 ± 0.08	1.52 ± 0.04	182.97 ± 5.42	2.95 ± 0.03	58	2.05 ± 0.09	74	0.98 ± 0.13	64
**13**	**4097**	10.18	4.91 ± 0.08	1.80 ± 0.03	0.62 ± 0.08	121.30 ± 5.04	2.29 ± 0.04	47	1.57 ± 0.05	87	0.52 ± 0.18	84
**14**	**4038**	9.46	4.49 ± 0.11	2.34 ± 0.03	1.13 ± 0.08	140.31 ± 0.24	2.57 ± 0.08	57	1.79 ± 0.01	76	1.00 ± 0.15	88
**15**	**4051**	12.98	4.79 ± 0.06	2.51 ± 0.14	0.85 ± 0.05	174.53 ± 7.21	2.57 ± 0.01	54	1.59 ± 0.05	63	0.60 ± 0.01	71
**16**	**5389**	10.40	3.91 ± 0.11	1.35 ± 0.08	0.64 ± 0.05	107.54 ± 0.91	1.56 ± 0.05	40	1.35 ± 0.08	>100	1.00 ± 0.06	>100

Table S1. *Cont.*

Sample	PRT 005 accession nº	Moisture content (%)	Whole Flour				Soaking Water					
			TPC_WF	TFC_WF	TPAC_WF	ORAC_WF	TPC_SW	TPC(SW)/ TPC(WF) (%)	TFC_SW	TFC(SW)/ TFC(WF) (%)	TPAC_SW	TPAC(SW)/T PAC(WF) (%)
						**Red varieties**						
**17**	**4120**	11.08	3.80 ± 0.05	1.24 ± 0.01	0.51 ± 0.11	97.72 ± 2.89	1.29 ± 0.06	34	0.90 ± 0.01	73	0.18 ± 0.03	35
**18**	**5387**	10.39	5.39 ± 0.16	1.52 ± 0.06	0.99 ± 0.09	151.03 ± 6.53	3.04 ± 0.07	56	1.48 ± 0.01	97	1.05 ± 0.09	>100
**19**	**4070**	10.13	4.44 ± 0.11	1.21 ± 0.04	0.07 ± 0.01	125.62 ± 3.28	1.78 ± 0.06	40	1.09 ± 0.03	90	0.49 ± 0.05	>100
**20**	**5382**	11.14	4.17 ± 0.10	1.49 ± 0.04	0.90 ± 0.13	90.82 ± 2.36	1.84 ± 0.04	44	1.19 ± 0.01	80	0.39 ± 0.04	43
**21**	**4149-R**	12.98	5.31 ± 0.12	1.79 ± 0.08	0.79 ± 0.05	162.13 ± 7.8	1.67 ± 0.06	31	1.02 ± 0.05	57	0.18 ± 0.01	23
						**Ligth brown variety**						
**22**	**4081**	12.98	4.99 ± 0.07	1.49 ± 0.01	0.48 ± 0.02	143.74 ± 6.8	3.31 ± 0. 10	66	1.86 ± 0.05	>100	0.43 ± 0.02	90
						**Brown varieties**						
**23**	**4182-B**	10.30	3.34 ± 0.09	0.89 ± 0.03	0.19 ± 0.03	80.80 ± 1.38	1.06 ± 0.02	32	0.56 ± 0.02	63	0.10 ± 0.02	53
**24**	**GC-34**	12.98	4.82 ± 0.02	1.55 ± 0.04	0.10 ± 0.01	171.13 ± 5.65	1.33 ± 0.01	28	1.11 ± 0.03	72	0.32 ± 0.02	>100
**25**	**GC-35**	12.98	4.05 ± 0.01	1.77 ± 0.05	0.56 ± 0.01	182.71 ± 5.37	2.22 ± 0.09	55	1.40 ± 0.05	79	0.19 ± 0.01	34
**26**	**GC-17**	12.98	4.72 ± 0.09	2.04 ± 0.01	0.52 ± 0.08	178.47 ± 5.24	1.74 ± 0.03	37	1.08 ± 0.01	53	0.28 ± 0.03	54
						**Brown background varieties with dark brown strips**						
**27**	**4194**	12.98	5.90 ± 0.08	2.88 ± 0.02	0.76 ± 0.04	188.73 ± 5.06	2.64 ± 0.07	45	1.84 ± 0.03	64	0.59 ± 0.07	78
**28**	**GC-40**	9.79	5.26 ± 0.30	1.51 ± 0.02	0.85 ± 0.02	138.89 ± 4.25	2.01 ± 0.03	38	1.14 ± 0.02	75	0.17 ± 0.04	20
**29**	**5384**	10.99	4.34 ± 0.07	1.33 ± 0.02	0.53 ± 0.04	103.91 ± 1.72	1.50 ± 0.05	35	0.85 ± 0.05	64	0.12 ± 0.03	23
**30**	**4085**	9.79	6.05 ± 0.17	2.23 ± 0.02	1.33 ± 0.13	146.58 ± 4.06	2.86 ± 0.10	47	1.61 ± 0.03	72	0.78 ± 0.09	59
**31**	**4071**	11.36	7.71 ± 0.35	2.78 ± 0.17	1.16 ± 0.23	213.36 ± 11.51	3.15 ± 0.08	41	2.10 ± 0.10	76	0.81 ± 0.05	70
**Average ± SD (n=31)**			4.08 ± 1.56	1.45 ± 0.83	0.52 ± 0.44	121.09 ± 52.10	1.65 ± 0.96		1.02 ± 0.63		0.37 ± 0.34	

Table S2. Average values ± Standard Deviation of Total Phenolic Content (TPC), mg GAE/g DW, Total Flavonoid Content (TFC), mg CE/g DW, and Total Proanthocyanidin Content (TPAC), mg CE/g DW, determined in soaked cotyledons (Cot) and coats (C)

Sample	PRT 005 accession nº	Cotyledons			Coats		
		TPC_Cot	TFC_Cot	TPAC_Cot	TPC_C	TFC_C	TPAC_C
				**White varieties**			
**1**	**4144**	0.93 ± 0.00	0.15 ± 0.00	0.01 ± 0.00	0.04 ± 0.00	0.01 ± 0.00	0.01 ± 0.00
**2**	**5383**	0.82 ± 0.02	0.15 ± 0.01	0.01 ± 0.00	0.04 ± 0.00	0.01 ± 0.00	0.01 ± 0.00
**3**	**4088**	0.88 ± 0.03	0.13 ± 0.00	0.01 ± 0.00	0.04 ± 0.00	0.01 ± 0.00	0.01 ± 0.00
**4**	**1979**	0.80 ± 0.04	0.14 ± 0.00	0.01 ± 0.00	0.03 ± 0.00	0.01 ± 0.00	0.01 ± 0.00
**5**	**5249**	0.92 ± 0.02	0.16 ± 0.00	0.01 ± 0.00	0.03 ± 0.00	0.01 ± 0.00	0.01 ± 0.00
				**Yellow variety**			
**6**	**5388**	0.85 ± 0.02	0.22 ± 0.00	0.01 ± 0.00	1.06 ± 0.06	0.49 ± 0.04	0.32 ± 0.01
				**White background variety with brown speckles and brown marginal pattern**			
**7**	**4185**	0.90 ± 0.05	0.17 ± 0.00	0.01 ± 0.00	1.80 ± 0.07	1.24 ± 0.04	1.10 ± 0.10
				**White and Brown background variety**			
**8**	**4189**	1.11 ± 0.04	0.18 ± 0.00	0.01 ± 0.00	2.96 ± 0.09	2.1 ± 0.09	1.39 ± 0.11
				**Pink varieties**			
**9**	**4110**	0.85 ± 0.03	0.18 ± 0.01	0.01 ± 0.00	3.92 ± 0.20	2.38 ± 0.28	3.47 ± 0.10
**10**	**4179**	1.03 ± 0.04	0.22 ± 0.00	0.01 ± 0.00	1.21 ± 0.04	0.77 ± 0.03	0.66 ± 0.14
				**Pink background variety with brown stripes**			
**11**	**4182-P**	1.02 ± 0.03	0.20 ± 0.00	0.01 ± 0.00	3.60 ± 0.03	2.29 ± 0.24	2.38 ± 0.45
				**Pink background varieties with purple stripes**			
**12**	**4119**	1.10 ± 0.03	0.24 ± 0.01	0.01 ± 0.00	3.86 ± 0.08	2.40 ± 0.16	2.28 ± 0.10
**13**	**4097**	1.01 ± 0.03	0.17 ± 0.00	0.01 ± 0.00	3.48 ± 0.06	2.21 ± 0.12	2.90 ± 0.74
**14**	**4038**	1.04 ± 0.02	0.21 ± 0.01	0.01 ± 0.00	4.07 ± 0.18	3.03 ± 0.06	2.51 ± 0.20
**15**	**4051**	1.15 ± 0.06	0.25 ± 0.00	0.01 ± 0.00	3.32 ± 0.14	2.20 ± 0.15	1.75 ± 0.25
**16**	**5389**	0.93 ± 0.03	0.20 ± 0.00	0.01 ± 0.00	3.93 ± 0.07	2.83 ± 0.19	4.00 ± 0.07

Table S2. *Cont.*

Sample	PRT 005 accession nº	Cotyledons			Coats		
		TPC_Cot	TFC_Cot	TPAC_Cot	TPC_C	TFC_C	TPAC_C
**Red varieties**							
**17**	**4120**	0.90 ± 0.03	0.18 ± 0.01	0.01 ± 0.00	3.47 ± 0.17	2.44 ± 0.25	1.96 ± 0.29
**18**	**5387**	1.12 ± 0.02	0.27 ± 0.01	0.01 ± 0.00	4.07 ± 0.19	2.37 ± 0.11	1.96 ± 0.35
**19**	**4070**	0.90 ± 0.00	0.21 ± 0.00	0.01 ± 0.00	3.56 ± 0.07	2.34 ± 0.12	1.84 ± 0.02
**20**	**5382**	0.86 ± 0.01	0.25 ± 0.00	0.01 ± 0.00	3.63 ± 0.10	2.49 ± 0.15	2.62 ± 0.31
**21**	**4149-R**	1.02 ± 0.07	0.22 ± 0.01	0.01 ± 0.00	4.13 ± 0.02	2.15 ± 0.16	3.50 ± 0.56
**Light Brown variety**							
**22**	**4081**	1.00 ± 0.01	0.22 ± 0.00	0.01 ± 0.00	3.14 ± 0.06	1.90 ± 0.10	1.98 ± 0.35
**Brown varieties**							
**23**	**4182-B**	0.91 ± 0.02	0.16 ± 0.01	0.01 ± 0.00	4.57 ± 0.07	2.50 ± 0.08	3.71 ± 0.69
**24**	**GC-34**	0.99 ± 0.02	0.19 ± 0.00	0.01 ± 0.00	3.60 ± 0.07	2.23 ± 0.23	1.86 ± 0.13
**25**	**GC-35**	1.07 ± 0.03	0.25 ± 0.00	0.01 ± 0.00	3.73 ± 0.31	2.33 ± 0.05	3.05 ± 0.13
**26**	**GC-17**	1.36 ± 0.06	0.29 ± 0.01	0.01 ± 0.00	4.61 ± 0.13	2.43 ± 0.23	2.29 ± 0.52
**Brown background varieties with dark brown stripes**							
**27**	**4194**	1.07 ± 0.02	0.29 ± 0.02	0.01 ± 0.00	3.72 ± 0.11	2.56 ± 0.18	3.23 ± 0.31
**28**	**GC-40**	1.03 ± 0.04	0.20 ± 0.01	0.01 ± 0.00	3.00 ± 0.18	2.08 ± 0.14	1.83 ± 0.22
**29**	**5384**	0.98 ± 0.00	0.19 ± 0.01	0.01 ± 0.00	2.74 ± 0.10	1.59 ± 0.09	2.07 ± 0.37
**30**	**4085**	1.33 ± 0.03	0.25 ± 0.01	0.01 ± 0.00	4.02 ± 0.18	2.93 ± 0.12	3.69 ± 0.18
**31**	**4071**	0.90 ± 0.00	0.20 ± 0.00	0.01 ± 0.00	3.96 ± 0.33	2.57 ± 0.16	2.25 ± 0.16
**Average ± SD (n=31)**		0.99 ± 0.13	0.20 ± 0.04	0.01 ± 0.00	2.88 ± 1.49	1.83 ± 0.97	1.96 ± 1.23

Table S3. Contribution of the different parameters analyzed in the two first principal components (* loadings ≥ |0.400|)

Matrices	Parameters	PC1	PC2
Whole Flour (WF)	TPC	0.636*	0.690*
	TFC	0.629*	0.674*
	Two step_TPAC	0.622*	0.641*
	ORAC	0.522*	0.731*
Soaking Water (SW)	TPC	0.539*	0.771*
	TFC	0.610*	0.717*
	LogTPAC	0.724*	0.562*
Soaked Cotyledons (Cot)	LogTPC	0.059	0.765*
	TFC	0.315	0.793*
Soaked Coats (C)	Two step_TPC	0.872*	0.250
	TFC	0.876*	0.294
	LogTPAC	0.724*	0.562*
% Total variance (81.7)		42.3	39.4

[1.] TPC, Total Phenolic Content; TFC, Total Flavonoid Content; TPAC, Total Proanthocyanidin Content; ORAC, Oxygen Radical Absorbance Capacity

Table S4. Average ± standard deviation values of the parameters analyzed in the different fractions, considering the clusters of common bean varieties defined by multivariate analysis. Different letters indicate values significantly different between clusters (Scheffé's test, $p < 0.05$)

Matrices	Parameters	Cluster 1	Cluster 2	Cluster 3
**Whole Flour (WF)**	**TPC (mg GAE/g DW)**	2.21 ± 1.10 **(a)**	5.10 ± 1.01 **(b)**	4.18 ± 0.88 **(b)**
	**TFC (mg CE/g DW)**	0.48 ± 0.41 **(a)**	1.84 ± 0.55 **(b)**	1.57 ± 0.76 **(b)**
	**TPAC* (mg CE/g DW)**	0.10 ± 0.15 **(a)**	0.65 ± 0.39 **(b)**	0.60 ± 0.27 **(b)**
	**ORAC (µmol TEAC/g DW)**	63.31 ± 35.71 **(a)**	151.26 ± 33.54 **(b)**	112.92 ± 42.94 **(b)**
**Soaking Water (SW)**	**TPC (mg GAE/g DW)**	0.49 ± 0.48 **(a)**	2.28 ± 0.66 **(c)**	1.50 ± 0.32 **(b)**
	**TFC (mg CE/g FW)**	0.25 ± 0.32 **(a)**	1.40 ± 0.39 **(b)**	1.00 ± 0.30 **(b)**
	**TPAC* (mg CE/g DW)**	0.05 ± 0.03 **(a)**	0.47 ± 0.28 **(b)**	0.44 ± 0.36 **(b)**
**Soaked Cotyledons (Cot)**	**TPC* (mg GAE/g DW)**	0.92 ± 0.10 **(a)**	1.04 ± 0.11 **(b)**	0.89 ± 0.03 **(a)**
	**TFC (mg CE/g DW)**	0.17 ± 0.03 **(a)**	0. 22 ± 0.04 **(b)**	0.20 ± 0.03 **(ab)**
**Soaked Coats (C)**	**TPC* (mg GAE/g DW)**	0.80 ± 1.05 **(a)**	3.58 ± 0.41 **(b)**	3.90 ± 0.42 **(b)**
	**TFC* (mg CE/g DW)**	0.52 ± 0.74 **(a)**	2.27 ± 0.31 **(b)**	2.53 ± 0. 18 **(b)**
	**TPAC (mg CE/g DW)**	0.39 ± 0.54 **(a)**	2.45 ± 0.68 **(b)**	3.15 ± 0.84 **(b)**
**Color**		• White (n=5)<br>• White background with brown speckles and brown marginal pattern (n=1)<br>• Yellow (n=1)<br>• White and brown background (n=1)<br>• Pink (n=1)	• Pink (n=1)<br>• Pink background with purple stripes (n=2)<br>• Red (n=2)<br>• Light brown (n=1)<br>• Brown (n=3)<br>• Brown background with dark brown stripes (n=5)	• Pink (n=1)<br>• Pink background with brown stripes (n=1)<br>• Red (n=2)<br>• Brown (n=1)

TPC, Total Phenolic Content; TFC, Total Flavonoid Content; TPAC, Total Proanthocyanidin Content; ORAC, Oxygen Radical Absorbance Capacity; *Differences established based on the results obtained for the transformed variables (Two-step TPAC_WF, LogTPC_Cot, Two-step TPC_C, Two-step TFC_C, LogTPAC_SW)

Table S5. Discriminant analysis to evaluate the clustering solution

Sample	PRT 005 accession nº	Probability Cluster 1	Probability Cluster 2	Probability Cluster 3
1	4144	**0.99969**	0.00030	0.00000
2	5383	**0.99983**	0.00017	0.00000
3	4088	**0.99967**	0.00032	0.00000
4	1979	**0.9999**	0.00010	0.00000
5	5249	**0.99995**	0.00005	0.00000
6	5388	**0.93889**	0.06106	0.00005
7	4185	**0.82926**	0.16890	0.00184
8	4189	**0.92679**	0.07316	0.00005
9	4110	0.00000	0.04910	**0.95090**
10	4179	**0.94335**	0.05665	0.00000
11	4182-P	0.04226	**0.9088**	0.04894
12*	4119	**-**	**-**	**-**
13	4097	0.00164	**0.95521**	0.04315
14*	4038	**-**	**-**	**-**
15	4051	0.00250	**0.9975**	0.00001
16	5389	0.00000	0.00391	**0.99609**
17	4120	0.01265	0.4774	**0.50995**
18	5387	0.00010	**0.99947**	0.00043
19	4070	0.01355	**0.89223**	0.09422
20	5382	0.00031	0.5045	**0.49519**
21	4149-R	0.00023	**0.81993**	0.17984
22	4081	0.00801	**0.99185**	0.00014
23	4182-B	0.00000	0.00017	**0.99983**
24	GC-34	0.01436	**0.9714**	0.01424
25	GC-35	0.00138	**0.99718**	0.00144
26*	GC-17	**-**	**-**	**-**
27	4194	0.00001	**0.99898**	0.00101
28	GC-40	0.03397	**0.96572**	0.0003
29	5384	0.22949	**0.7686**	0.00191
30	4085-P	0.00000	**0.99668**	0.00332
31	4071	0.00000	**0.73148**	0.26852

*Excluded samples from multivariate analysis (values of TPC_C, TFC_C and TPAC_WF were out of ranking in the two-step transformed variables)

Table S6. Molecular structure of the compounds identified

Family	Molecular structure	Substituent groups	Compound
**Hydroxybenzoic acids**	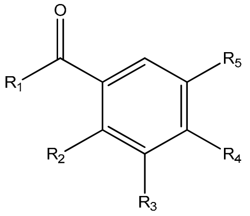	**R_1**-OH; **R_2**-H; **R_3**-OH; **R_4**-OH; **R_5**-H	Protocatechuic acid
		**R_1**-OH; **R_2**-H; **R_3**-OH; **R_4**-$OC_6H_{12}O_6$; **R_5**-H	Protocatechuic acid-4-*O*-glucoside
		**R_1**-OH; **R_2**-H; **R_3**-H; **R_4**-OH; **R_5**-H	*p*-hydroxybenzoic acid
		**R_1**-OH; **R_2**-H; **R_3**-H; **R_4**-$OC_6H_{12}O_6$; **R_5**-H	*p*-hydroxybenzoic acid-4-*O*-glucoside
		**R_1**-OH; **R_2**-H; **R_3**-H; **R_4**-OH; **R_5**-OCH_3	Vanillic acid
		**R_1**-OH; **R_2**-H; **R_3**-H; **R_4**-OH; **R_5**-OH	Gentisic acid
**Hydroxycinnamic acids**	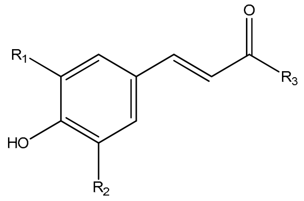	**R_1**-OCH_3; **R_2**-H; **R_3**-OH	Ferulic acid
		**R_1**-H; **R_2**-H; **R_3**-OH	*p*-Coumaric acid
		**R_1**-OCH_3; **R_2**-OCH_3; **R_3**-OH	Sinapic acid
		**R_1**-OCH_3; **R_2**-H; **R_3**-$C_6H_{10}O_8$	trans-Feruloyl aldaric acid isomers
		**R_1**-H; **R_2**-H; **R_3**-$C_6H_{10}O_8$	trans-*p*-coumaroyl aldaric acid isomers
		**R_1**-OCH_3; **R_2**-OCH_3; **R_3**-$C_6H_{10}O_8$	Sinapoyl aldaric acid isomers
**Flavan-3-ol**	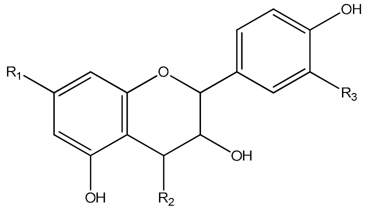	**R_1**-OH; **R_2**-H; **R_3**-OH	(+)-Catechin/ (-)-Epicatechin
		**R_1**-OH; **R_2**-H; **R_3**-$OC_6H_{12}O_6$	(+)-Catechin-3′-*O*-glucoside
		**R_1**-$OC_6H_{12}O_6$; **R_2**-H; **R_3**-OH	(+)-Catechin-7-*O*-glucoside
		**R_1**-OH; **R_2**-$C_{15}H_{14}O_6$; **R_3**-OH	Procyanidin B1, B2, B3, B4, B5
		**R_1**-OH; **R_2**-$C_{30}H_{26}O_{12}$; **R_3**-OH	Procyanidin C1, C2
**Flavanone**	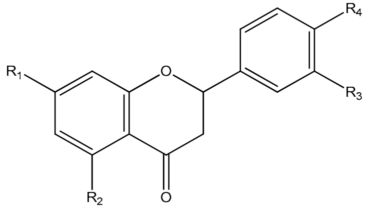	**R_1**-OH; **R_2**-OH; **R_3**-OH; **R_4**-OH	Eriodictyol (–R groups, could be substituted by –$OC_6H_{12}O_6$ giving rise to 4 different eriodictyol-*O*-hexoside compounds)
		**R_1**-OH; **R_2**-OH; **R_3**-H; **R_4**-OH	Naringenin
		**R_1**-$OC_6H_{12}O_6$; **R_2**-OH; **R_3**-H; **R_4**-OH	Naringenin-7-*O*-glucoside

Table S3. *Cont.*

Family	Molecular structure	Substituent groups	Compound
**Flavonols**	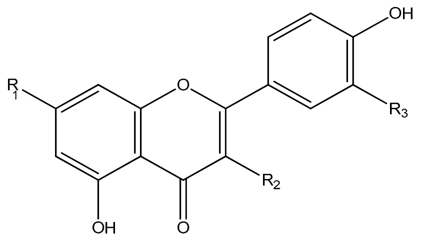	**R**$_1$-OH; **R**$_2$-OH; **R**$_3$-H	Kaempferol
		**R**$_1$-OH; **R**$_2$-$OC_{11}H_{20}O_{10}$; **R**$_3$-H	Kaempferol-3-*O*-xylosyl-glucoside
		**R**$_1$-OH; **R**$_2$-$OC_6H_{12}O_6$; **R**$_3$-H	Kaempferol-3-*O*-glucoside
		**R**$_1$-OH; **R**$_2$-$OC_8H_{14}O_7$; **R**$_3$-H	Kaempferol-3-*O*-acetyl-glucoside
		**R**$_1$-OH; **R**$_2$-OH; **R**$_3$-OH	Quercetin
		**R**$_1$-OH; **R**$_2$-$OC_6H_{12}O_6$; **R**$_3$-OH	Quercetin-3-*O*-glucoside
		**R**$_1$-OH; **R**$_2$-$OC_8H_{14}O_7$; **R**$_3$-OH	Quercetin-3-(6-*O*-acetyl-*β*-glucoside)
		**R**$_1$-$OC_6H_{12}O_6$; **R**$_2$-H; **R**$_3$-$OC_6H_{12}O_6$	Luteolin-3′,7-di-*O*-glucoside
**Isoflavone**	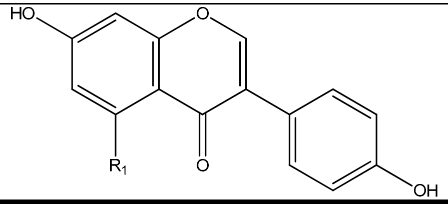	**R**$_1$-H	Daidzein
		**R**$_1$-OH	Genistein
